# Single Cell Mass Cytometry Revealed the Immunomodulatory Effect of Cisplatin Via Downregulation of Splenic CD44+, IL-17A+ MDSCs and Promotion of Circulating IFN-γ+ Myeloid Cells in the 4T1 Metastatic Breast Cancer Model

**DOI:** 10.3390/ijms21010170

**Published:** 2019-12-25

**Authors:** József Á. Balog, László Hackler Jr., Anita K. Kovács, Patrícia Neuperger, Róbert Alföldi, Lajos I. Nagy, László G. Puskás, Gábor J. Szebeni

**Affiliations:** 1 Laboratory of Functional Genomics, Biological Research Centre, Temesvári krt. 62, H6726 Szeged, Hungary; balog.jozsef@brc.hu (J.Á.B.); neuperger.patricia@brc.hu (P.N.); laszlo@avidinbiotech.com (L.G.P.); 2 PhD School in Biology, University of Szeged, H6726 Szeged, Hungary; r.alfoldi@astridbio.com; 3 AstridBio Technologies Ltd., Also kikötő sor 11/D, H6726 Szeged, Hungary; laszlo.hackler@astridbio.com; 4 Avidin Ltd., Also kikötő sor 11/D, H6726 Szeged, Hungary; a.kovacs@avidinbiotech.com (A.K.K.); l.nagy@avidinbiotech.com (L.I.N.); 5 Department of Physiology, Anatomy and Neuroscience, Faculty of Science and Informatics, University of Szeged, Közép fasor 52, H6726 Szeged, Hungary

**Keywords:** single cell mass cytometry, metastatic breast cancer, myeloid-derived suppressor cells, immunophenotyping

## Abstract

The treatment of metastatic breast cancer remained a challenge despite the recent breakthrough in the immunotherapy regimens. Here, we addressed the multidimensional immunophenotyping of 4T1 metastatic breast cancer by the state-of-the-art single cell mass cytometry (CyTOF). We determined the dose and time dependent cytotoxicity of cisplatin on 4T1 cells by the xCelligence real-time electronic sensing assay. Cisplatin treatment reduced tumor growth, number of lung metastasis, and the splenomegaly of 4T1 tumor bearing mice. We showed that cisplatin inhibited the tumor stroma formation, the polarization of carcinoma-associated fibroblasts by the diminished proteolytic activity of fibroblast activating protein. The CyTOF analysis revealed the emergence of CD11b+/Gr-1+/CD44+ or CD11b+/Gr-1+/IL-17A+ myeloid-derived suppressor cells (MDSCs) and the absence of B220+ or CD62L+ B-cells, the CD62L+/CD4+ and CD62L+/CD8+ T-cells in the spleen of advanced cancer. We could show the immunomodulatory effect of cisplatin via the suppression of splenic MDSCs and via the promotion of peripheral IFN-γ+ myeloid cells. Our data could support the use of low dose chemotherapy with cisplatin as an immunomodulatory agent for metastatic triple negative breast cancer.

## 1. Introduction

The role of the tumor microenvironment, the interaction of cancer cells with the extracellular matrix, endothelial cells, cancer-associated fibroblasts, and leukocytes in the tumor stroma have been increasingly considered as a milestone in cancer development, especially in the last decade [1,2]. The deeper understanding of the disturbances in the regulation and activation of the immune system in cancer resulted in the advancement of anti-cancer therapies, such as the immune checkpoint blockade (ICB) [3]. However, the treatment of poorly immunogenic and metastasizing tumors remained a challenge.

Here, we focus on female breast cancer since it is the most frequent cancer in women and still the deadliest cancer type between the ages of 20–49 years old in contrast to the achievements in early diagnostics and therapeutics [4,5]. In our work the murine mammary carcinoma of the BALB/c mice, the syngeneic 4T1 was studied [6]. The 4T1 model is among the few murine triple negative breast cancer (TNBC) models that spontaneously metastasize to sites affected in human breast cancer (e.g., lung) in an immunocompetent host [7]. Orthotopic transplantation of 4T1 cells offers a relevant tumor model to study efficacy of drug candidates or immune therapy regimens [8]. Previously, we showed the tumor promoting effect of mesenchymal stem cell (cancer associated fibroblast)-derived galectin-1 in the 4T1 model [9], and later on we screened an anti-cancer compound library of imidazo[1-2-b]pyrazole-7-carboxamides in both two- and three-dimensional cell cultures of 4T1 cells [10,11].

We have previously reviewed how cancer-related chronic inflammation can lead to the generation of immature myeloid-derived suppressor cells (MDSCs) and to the alternative polarization of tumor-associated macrophages (TAMs) [12], which manifests autonomously in the 4T1 breast cancer model [13,14]. It has been shown that the granulocytic MDSCs support metastases by suppressing CD8+ T-cells in the 4T1 breast tumor model [15]. It was also recently shown that 4T1 cells shape immune responses with an increase of splenic CD11b+ cells to promote cancer growth in an *Shb* (SRC homology-2 domain protein B) dependent manner [16]. The 4T1 tumor cells are poorly immunogenic and refractory to immune therapies, although the combination of anti-PD-1, anti-CTLA-4 ICB with epigenetic modulators could have a therapeutic benefit curing more than 80% of 4T1 tumor bearing mice via eliminating MDSCs [17]. We have previously reviewed strategies targeting these myeloid-derived suppressors cells or tumor associated macrophages to combat cancer [18]. Here, the traditional chemotherapeutic agent, the DNA crosslinker cisplatin was used, since cisplatin and platinum-based chemoterapeutics are in the clinical routine as first line treatment option in several cancers such as lung, bladder, ovarian and metastatic breast cancer [19]. Recent studies have shown the immune induction by cisplatin in human TNBC (the TONIC trial NCT02499367) [20], or in murine carcinoma models showing enhanced sensitivity to ICB therapy in combination with cisplatin treatment but these studies did not deal with immunophenotyping of the myeloid compartment [21,22]. The beneficial effect of cisplatin on the course of 4T1 tumor development was shown recently in combination with metformin or bromelain [23,24], but these studies also did not address the characterization of the immunophenotype.

To the best of our knowledge our study is the first, where mass cytometry, a multidimensional single cell technology with computational data analysis was carried out in order to reveal the immunophenotype of 4T1 murine triple negative breast carcinoma and the effect of cisplatin treatment on the splenic and circulating immune compartments.

## 2. Results

### 2.1. Real-Time Monitoring of 4T1 Cell Viability Hampered by Cisplatin

**Figure 1:**
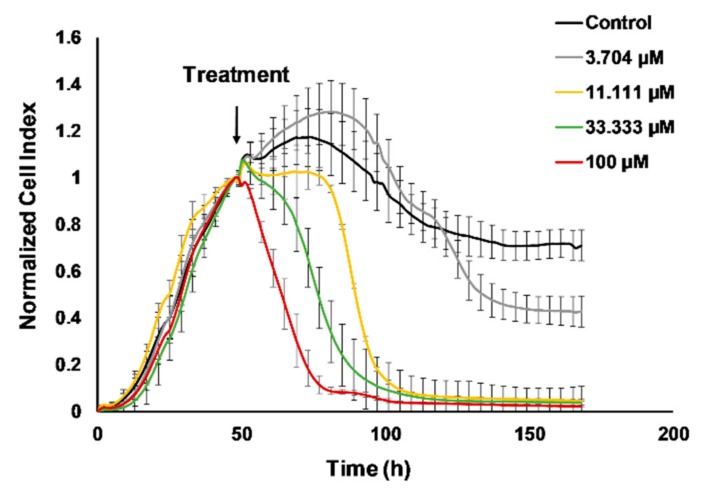
Real-time monitoring of 4T1 cell viability hampered by cisplatin. The 4T1 cells were seeded and the baseline impedance was recorded for 48 h. After that 48 h culturing treatment with 11.111 µM, 33.333 µM, or 100 µM cisplatin reduced viability of 4T1 cells on a time and dose dependent manner. The corresponding dose-response curves with the half maximal inhibitory concentration (IC_50_) values can be found in Figure S1.

Determination of the half maximal inhibitory concentration, the IC_50_ of cisplatin on 4T1 cells was carried out using the real-time electronic sensing xCelligence system [25]. The detected impedance is proportional with the percentage of adhered living cells to the gold coated plate and the decline in the normalized cell index corresponds to hampered cell viability (Figure 1). The effect of cisplatin on viability was followed for 120 h after treatment in every 15 min (former studies reported endpoint assays with cisplatin on 4T1 cells). The IC_50_ values were as follows 36.74 µM at 24 h, 7.608 µM at 48 h, 6.962 µM at 72 h, 4.128 µM at 96 h, and 3.995 µM at 120 h (Figure S1).

### 2.2. Cisplatin Treatment Reduced 4T1 Tumor Growth, the Number of Lung Metastatic Nodules and the Weight of the Spleen

**Figure 2:**
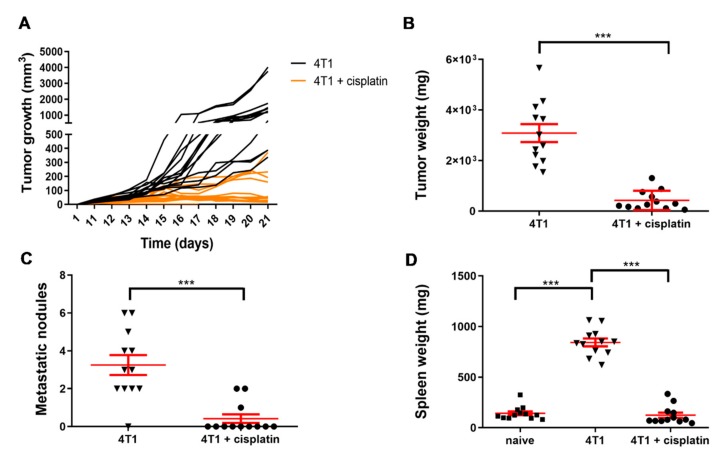
Cisplatin treatment reduced 4T1 tumor growth, the number of lung metastatic nodules and the weight of the spleen. The 4T1 cells (1.2 × 10^5^) were transplanted by the injection into the mammary fat pad of BALB/c mice (*n* = 12). Tumor growth was monitored daily (**A**). On the 23rd day mice were euthanized and the weight of the tumors (**B**), the number of metastatic nodules on the lungs (**C**), and the weight of the spleens were measured (**D**). Individual values and arithmetic mean values of the samples ± standard error of the mean (SEM) are plotted, statistical significance was set to *** *p* < 0.001.

The syngeneic BALB/c mice were orthotopically transplanted with 4T1 breast cancer cells in order to establish the animal model for the addressed immunophenotyping. Tumor growth was monitored daily. All mice treated with cisplatin showed markedly reduced tumor growth compared to untreated 4T1 tumor bearing mice represented by the average tumor volume of 102 mm^3^ versus (vs.) 1481 mm^3^ on the 21st day (Figure 2A). On the 23rd day mice were euthanized for immunophenotyping and the weight of the tumors (Figure 2B), the number of metastatic nodules (macrometastasis) on the lungs (Figure 2C), and the weight of the spleens were measured (Figure 2D).

The average weight of the tumors was reduced by almost 90% due to cisplatin treatment, namely 426.4 ± 110.1 mg (mean ± SEM) in the cisplatin treated 4T1 tumorous mice vs. 3087 ± 356 mg in the untreated 4T1 tumor bearing mice (Figure 2B). The development of metastatic nodules on the surface of the lungs were also inhibited by cisplatin, the average number of lung macrometastasis were as follows: 0.42 ± 0.23 in cisplatin treated vs. 3.25 ± 0.524 in untreated 4T1 tumorous mice (Figure 2C). Splenomegaly is one sign of myeloid cell expansion due to cancer related inflammation in tumor bearing hosts [26]. Cisplatin treatment suppressed the enlargement of the spleen as spleen weights were 143.04 ± 19.25 mg, 843.59 ± 38.69 mg, 123.91 ± 26.08 mg, in the naive, 4T1 tumor bearing and cisplatin treated 4T1 tumor bearing mice, respectively (Figure 2D).

### 2.3. Cisplatin Reduced the Activity of Fibroblast Activator Protein (FAP)

**Figure 3:**
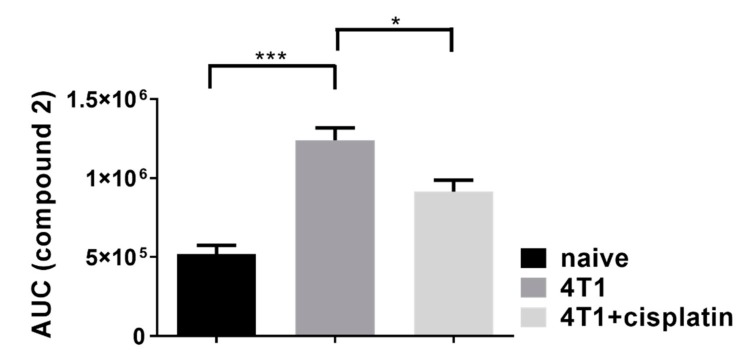
The proteolytic activity of the fibroblast activator protein (FAP) increased by the formation of breast cancer, and was significantly decreased by cisplatin treatment. Area under the curve (AUC) values from high pressure liquid chromatography (HPLC) analysis of the peptide digestion product **2** (Figure S2) were plotted. The results are shown as arithmetic mean values of the samples ± standard error of the mean (SEM), statistical significance was set to *** *p* < 0.001, * *p* < 0.05.

The increased activity of the prolyl endopeptidase FAP enzyme is a hallmark of the tumor stroma, because of the accumulation and activation of cancer associated fibroblasts (CAFs). The FAP protease activity either membrane bound on CAFs or solubilized, is proportional with the malignancy [27]. The activity of FAP enzyme was investigated, because it has been reported that the accumulation of CAFs in the tumor stroma and their expression of FAP contributes to the chemoresistance to cisplatin in carcinomas [28,29]. We have synthetized a peptide substrate (Fmoc-Gly-Pro-Cysteic acid-Ile-Gly-NH_2_, Figure S2) in order to measure FAP activity in the plasma of naive, 4T1 tumor bearing and cisplatin treated 4T1 tumorous mice (Figure 3).

FAP enzyme activity quantification was based on a direct measurement of a digested peptide product with high pressure liquid chromatography (HPLC) (Figure S2). Fmoc-GP-Cox- was synthesized (Avidin Ltd.) and used as a substrate, where “Cox” denotes for oxydized cysteine, namely, cysteic acid. We used oxidized cysteine amino acid at position 3 in the peptide substrate of FAP, instead of unmodified cysteine. In a preliminary experiment, we confirmed that the peptide with the oxidized cysteine residue was the same or better substrate of FAP. Moreover, our improved peptide substrate is more water soluble and after FAP digestion the dipeptide product (Fmoc-GP) could be better separated from the intact substrate during HPLC analysis, then the unmodified version, enabling us more accurate and sensitive quantification. We could detect FAP activity in the plasma of naive, 4T1 tumor bearing and cisplatin treated 4T1 tumorous animals with the following average AUC (± SEM) values from HPLC analysis: 518.5 ± 55.5 × 103, 1240.2 ± 77.9 × 103, 914.9 ± 71.7 × 103, respectively (Figure 3).

### 2.4. Single Cell Mass Cytometry (SCMC) Revealed the Immunophenotype of Breast Cancer Bearing Mice

#### 2.4.1. Cisplatin Restored the Splenic Immunophenotype of 4T1 Tumor Bearing Mice

**Figure 4:**
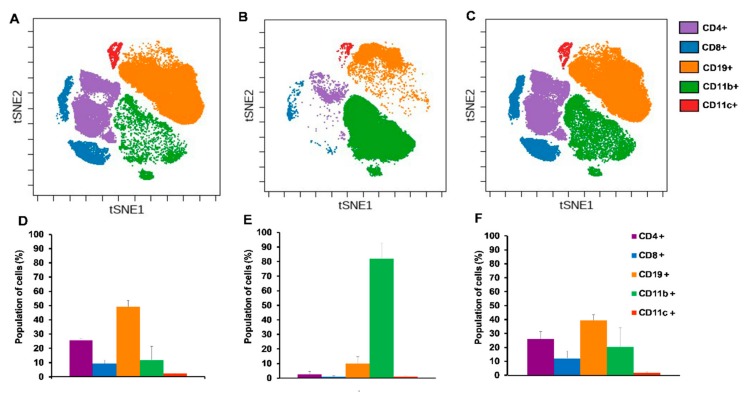
Single cell mass cytometric immunophenotyping showed the accumulation of CD11b+ myeloid cells in 4T1 tumor bearing animals at the expense of lymphoid subsets which was reverted by cisplatin treatment. The visualization of stochastic neighbor embedding (viSNE) analysis was run on the (**A**) naive, (**B**) 4T1 tumor bearing, and (**C**) cisplatin treated 4T1 tumorous animals within the CD45+ living singlets. Quantitative analysis of the main lymphoid subsets: CD4+ T-cells = lilac, CD8+ T-cells = blue, CD19+ B-cells = orange and the myeloid CD11b+ = green, CD11c + = red subsets were performed in the spleen of (**D**) naive, (**E**) 4T1 tumor bearing, and (**F**) cisplatin treated 4T1 tumorous mice within the CD45+ living singlets. Representative viSNE plots and column bars are shown from the pooled samples of 6 mice per group. Data are shown as arithmetic means on the column bars ± SEM. Pairwise comparison of the emergence of CD11b+ population in (**E**) vs. (**D**) and its decrease by cisplatin treatment (**F**) vs. (**E**) has statistical significance at *p* < 0.001.

In order to investigate the immunophenotype of 4T1 breast cancer single cell mass cytometry was performed with 24 antibodies in one single tube (see Table 1 in Section 4.5). We intended to monitor the draining lymph nodes, bone marrow, spleen, and blood of the naive, 4T1 tumor bearing, and cisplatin treated 4T1 tumorous animals. However, the orthotopic injection of 4T1 cells into the mammary fat pad resulted in the outgrowth of the tumor mass in co-junction with the draining cervical and axillary lymph nodes making them undetectable. Bone marrow staining showed homogenous immature cells (little CD11+ Gr1+ elevation in tumor bearing hosts) excluding these samples from further analysis. Our attention turned toward the spleen since it has been published in the seminal paper of Bronte et al. that tolerance to tumor antigens develops in the spleen [30]. During the SCMC analysis of the spleen CD45+ living singlets were gated and on these leukocytes the unsupervised and multidimensional visualization of stochastic neighbor embedding (viSNE) analysis was performed delineating the separate clouds of different main immune subsets (Figure 4) [31].

Both the CD4+ and CD8+ T-cells were almost completely absent and the percentage of CD19+ B-cells decreased from 50% to 10% of CD45+ living singlets in the spleen of the 4T1 tumor bearing mice (Figure 4D,E). The percentage of the myeloid CD11b+ cells increased from 10% to 80% at the expense of lymphoid subsets due to breast cancer development (Figure 4D,E). Cisplatin treatment of 4T1 tumorous mice normalized the splenic immunophenotype similar to naive mice with 25% CD4+, 10% CD8+ T-cells, 40% CD19+ B-cells, and 20% CD11b+ cells (Figure 4F).

**Figure 5:**
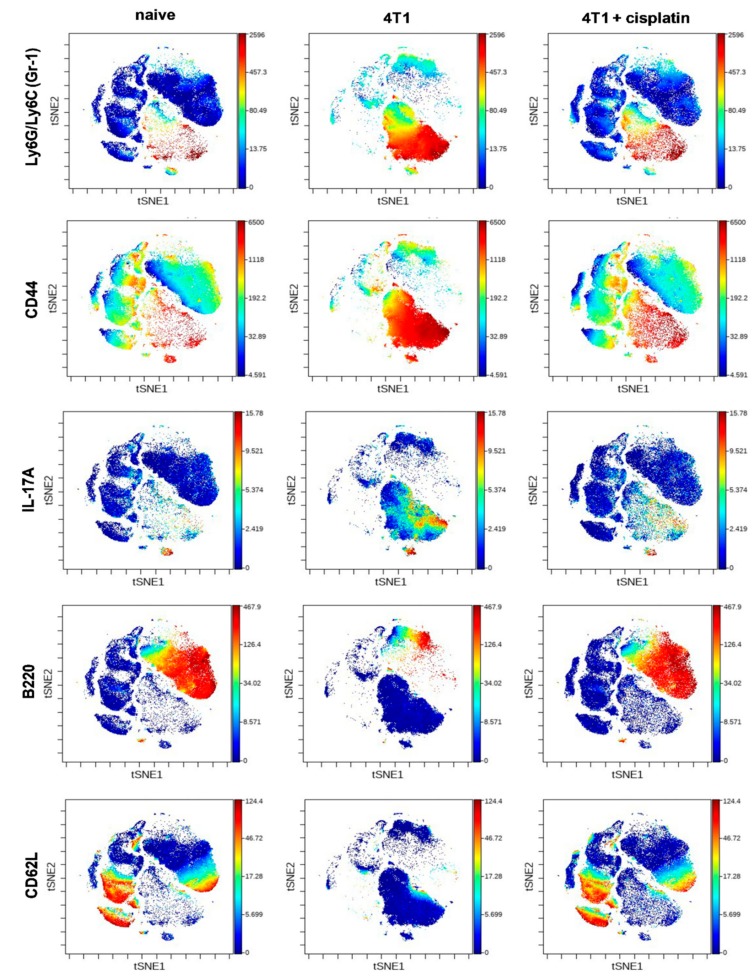
The viSNE plots illustrate the expression intensity of Gr-1, CD44, IL-17A, B220, CD62L markers within the clouds of main subsets defined in Figure 4 in the splenic samples of naive, 4T1 tumor bearing, and cisplatin treated 4T1 tumorous mice. The coloration is proportional to the expression intensity (blue = low, red = high). The list of the antibodies can be found in Table 1 in Section 4.5. Representative viSNE plots are shown from the pooled samples of 6 mice per group. The markers of the panel which were not detected or did not show differential expression are not shown.

The expression intensity of certain proteins within the viSNE plots of main subsets showed dramatic changes (Figure 5). The expression pattern of Gr-1+ (Ly6C/Ly6G), CD44+, and IL-17A+ cells within the cloud of CD11b+ myeloid cells were uniquely high in the spleen of 4T1 breast cancer bearing mice (Figure 5) in accordance with the splenomegaly shown in Figure 2D. On the contrary, the B220 and CD62L markers within the cloud of CD19+ B-cells, and the CD62L within the cloud of CD4+ and CD8+ T-cells also were highly reduced in the spleen of 4T1 tumorous mice (Figure 5). Cisplatin treatment reverted the immunophenotype of both splenic CD11b+ myeloid (Gr-1+, CD44+ and IL-17A+) cells and lymphoid (B220+/CD19+, CD62L+/CD19+, CD62L+/CD4+, CD62L+/CD8+ T-cells) cells similar to naive mice (Figure 5).

**Figure 6:**
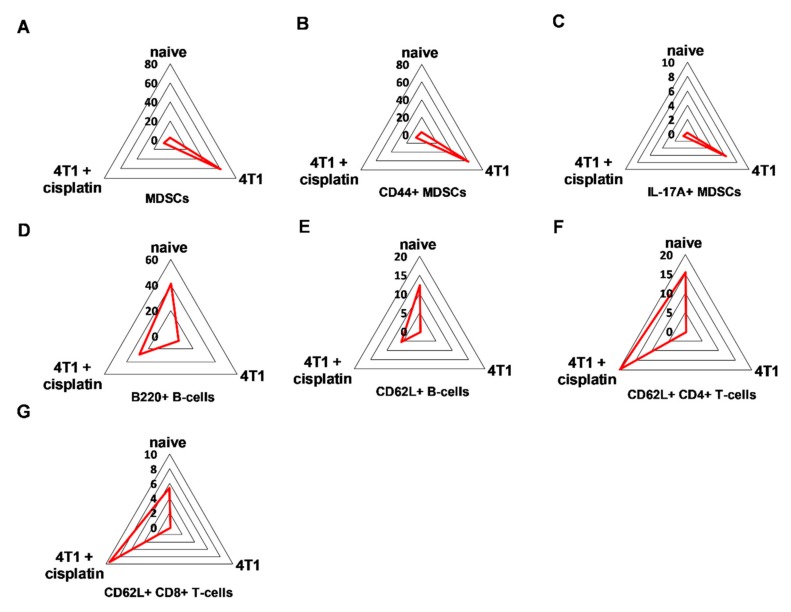
The trajectories on the radar plots delineate the characteristic marker profile of splenocytes in naive, 4T1 tumor bearing, and cisplatin treated tumorous mice. The accumulation of splenic (**A**) CD11b+/Gr-1+ MDSCs, (**B**) CD44+, and in a smaller extent (**C**) IL-17A+ MDSCs is a characteristic of 4T1 breast cancer. Cisplatin restores the percentage of (**D**) B220+ and (**E**) CD62L+ B-cells, (**F**) CD62L+ CD4+ and (**G**) CD8+ T-cells. The percentage of the given populations is demonstrated on the radar plots within the CD45+ living singlets determined by manual gating in Cytobank. The gating hierarchy is explained in the text and in Figure S3. The markers of the panel which were not detected or did not show differential expression are not shown. Representative radar plots are shown from the pooled samples of 6 mice per group as described in Section 4.5.

Quantitation of the populations with characteristic protein expression was performed by manual gating within the CD45+ living singlets of splenocytes (Figure S3). The trajectories on the radar plots delineate the characteristic marker profile of splenocytes of naive, 4T1 tumor bearing, and cisplatin treated tumorous mice (Figure 6).

MDSCs were defined as CD45+/CD3-/CD11+/Gr1+ cells and further evaluated for CD44 and IL-17A staining. These MDSCs, CD44+ MDSCs and IL-17A+ MDSCs represented exclusively 60%, 60% and 6% of CD45+ living singlets in the spleen of 4T1 tumorous mice, respectively. The B-cell marker B220 (except regulatory B-cells, germinal center B-cells, some plasma cells, and certain memory B-cells [32,33,34]) on CD45+/CD3-/CD19+ B-cells was 40% in the naive, 30% in the cisplatin treated, while only 6% in the untreated 4T1 tumorous mice. The homing receptor L-selectin, CD62L was almost absent (0.2%) on the splenic B-cells of 4T1 tumorous mice but it was restored upon cisplatin treatment to 5.5% (naive mice 12.5%). The CD45+/CD3+/TCRβ+/CD4+ and CD45+/CD3+/TCRβ+/CD8+ T-cells expressed CD62L 15% vs. 20% and 6% vs. 10% in the splenocytes of naive and cisplatin treated 4T1 bearing mice, respectively. Sunburst charts represent the immunocomposition of main subsets of the spleens detected by single cell mass cytometry in Figure S4A and FlowSOM (Flow data using Self-Organizing Map, [35]) analysis demonstrates the identified clusters, minimum spanning trees (MSTs) of splenic subsets in Figure S5.

#### 2.4.2. Cisplatin Could not Completely Restore the Peripheral Immunophenotye of 4T1 Tumor Bearing mice but Increased IFN-γ Production of Myeloid Cells

**Figure 7:**
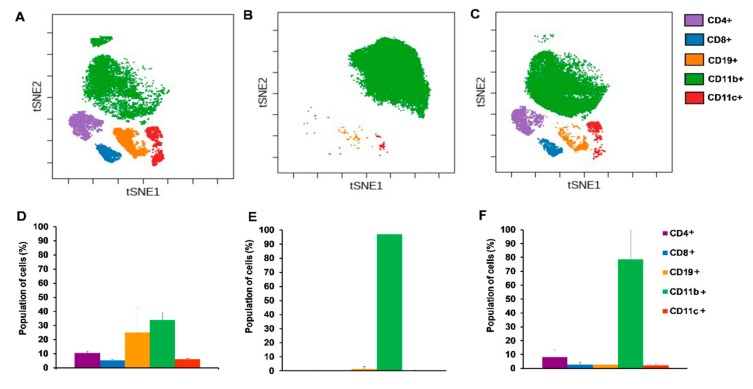
Immunophenotyping of blood showed dramatic expansion of CD11b+ cells in advanced cancer. The viSNE analysis was run on the (**A**) naive, (**B**) 4T1 tumor bearing, and (**C**) cisplatin treated 4T1 tumorous animals within the CD45+ living singlets. Quantitative analysis of the main lymphoid subsets: CD4+ T-cells = lilac, CD8+ T-cells = blue, CD19+ B-cells = orange and the myeloid CD11b+ = green, CD11c + = red subsets was performed in the blood of (**D**) naive, (**E**) 4T1 tumor bearing, and (**F**) cisplatin treated 4T1 tumorous mice within the CD45+ living singlets. Representative viSNE plots and column bars are shown from the pooled samples of 6 mice per group. Data are shown as arithmetic means on the column bars ± SEM. Pairwise comparison of the emergence of CD11b+ population in (**E**) vs. (**D**) has significance at *p* < 0.001.

Immunophenotype of the blood in breast cancer can reflect the state of the peripheral tolerance associated with cancer-related inflammation or the activation of anti-tumor effector cellular responses [36]. The viSNE plots of the main subsets in blood samples show the absence of peripheral CD4+, CD8+ T-, and B-cells and the emergence of CD11b+ cells as a sign of advanced cancer compared to naive blood (Figure 7A,B). Cisplatin treatment at least partially normalized the immunocomposition of the blood of 4T1 tumorous mice (Figure 7C).

The percentage of CD11b+ cells of 4T1 tumor bearing mice due to advanced cancer-related myeloid expansion represented around 97% (Figure 7E) in contrast to naive mice with 35% CD11b+ cells (Figure 7D) of CD45+ living singlets in the blood. The treatment of 4T1 breast cancerous mice with cisplatin could not suppress the emergence of CD11b+ cells significantly with an average of 85% CD11b+ cells (Figure 7F). Myeloid expansion led almost to the absence of circulating T- and B-lymphocytes in advanced cancer (Figure 7E). Cisplatin treatment at least partially restored the percentage of these CD4+, CD8+ T- and CD19+ B-lymphocytes with an average 8.1% vs. 10.5%, 2.6% vs. 5.1%, 2.7% vs. 24.8% in naive mice, respectively (Figure 7D,F).

**Figure 8:**
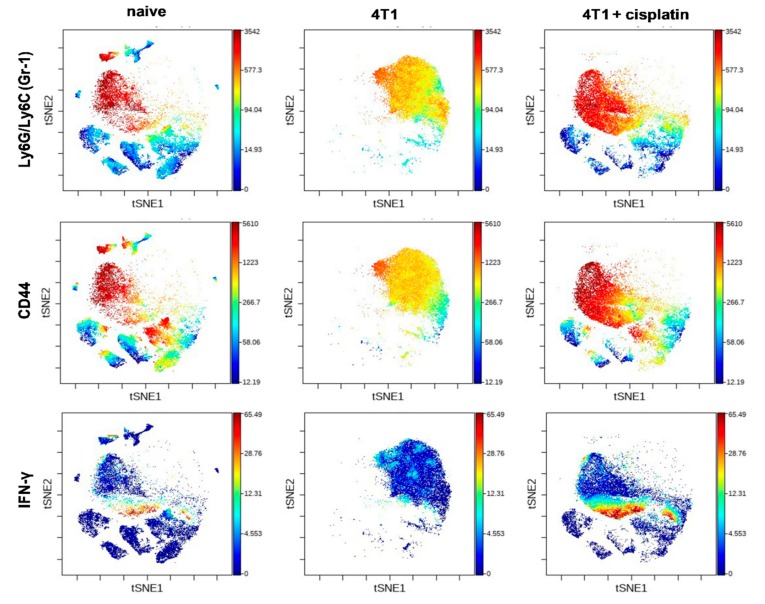
The viSNE plots illustrate the expression intensity of Gr-1, CD44, and IFN-γ markers within the clouds of main subsets defined in Figure 7 in the blood samples of naive, 4T1 tumor bearing, and cisplatin treated 4T1 tumorous mice. The coloration is proportional with the expression intensity (blue = low, red = high). The list of the antibodies can be found in Table 1 in the Section 4.5. Representative viSNE plots are shown from the pooled samples of 6 mice per group. The markers of the panel which were not detected or did not showed different expression are not shown.

The differential expression intensity of the Gr-1, CD44, IFN-γ markers on blood-derived leukocytes was plotted on the viSNE graphs (Figure 8). Interestingly, the highest expression protein pattern of Gr-1+/CD44+ (Gr-1+ ^bright^/CD44+ ^bright^) within the CD11b+ subset was associated with early cancer disease in the blood of cisplatin treated 4T1 tumorous mice. The Gr-1+ ^dim^/CD44+ ^dim^ immature myeloid cells have been reported to suppress T-cells and IFN-γ production [37,38], the dim expression intensity of these markers was characteristic to 4T1 tumor bearing mice (Figure 8). In line with this, the smaller population of IFN-γ producing myeloid cells appeared within the CD11 subtype by cisplatin treatment which was completely absent in the advanced cancer (Figure 8).

**Figure 9:**
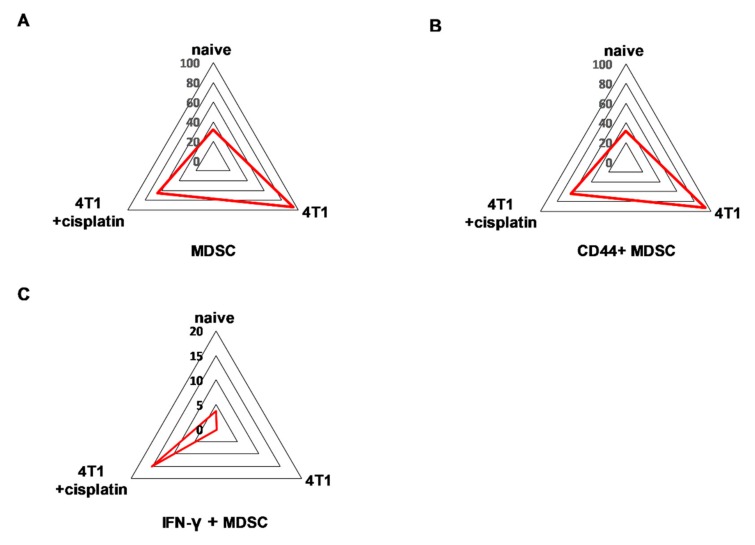
The trajectories on the radar plots delineate the characteristic marker profile of blood-derived leukocytes in naive, 4T1 tumor bearing, and cisplatin treated tumorous mice. The accumulation of peripheral (**A**) CD11b+/Gr-1+ MDSCs and (**B**) CD44+ MDSCs is a characteristic of 4T1 breast cancer. (**C**) Due to cisplatin treatment IFN-γ+ MDSCs were developed at the periphery. The percentage of the given populations is demonstrated on the radar plots within the CD45+ living singlets determined by manual gating in Cytobank. The gating hierarchy is explained in the text. The markers of the panel which were not detected or did not show differential expression are not shown. Representative radar plots are shown from the pooled samples of 6 mice per group as described in Section 4.5.

Quantitation of the populations with characteristic protein expression was performed by manual gating within the CD45+ living singlets of blood-derived leukocytes. The trajectories on the radar plots delineate the characteristic marker profile of peripheral leukocytes of naive, 4T1 tumor bearing, and cisplatin treated tumorous mice (Figure 9).

MDSCs were defined as CD45+/CD3-/CD11+/Gr1+ cells (Figure 9A) and further evaluated for the expression of CD44 (Figure 9B) and IFN-γ (Figure 9C). The total MDSCs, CD44+ MDSCs and IFN-γ + MDSCs represented 92%, 93%, and only 0.15% of CD45+ living singlets in the spleen of 4T1 advanced tumorous mice, respectively. Administration of cisplatin resulted in the decrease of the percentage of both MDSCs, CD44+ MDSCs to 63% (naive blood 32%). Interestingly, IFN-γ which is indispensable for antitumor immune response [39], was increased by cisplatin in 15% of MDSCs (naive 3%). Sunburst charts represent the immunocomposition of main subsets of the blood detected by single cell mass cytometry in Figure S4B and FlowSOM (Flow data using Self-Organizing Map, [35]) analysis demonstrates the identified clusters, minimum spanning trees (MSTs) of peripheral subsets in Figure S5.

## 3. Discussion

Solid tumors manifest when cancer cells escape from immunosurveillance. During cancer development, malignant cells develop strategies to induce peripheral tolerance and in parallel inflammatory cells change their phenotype to nurse the tumor and suppress anti-tumor effector functions [36,40]. Since metastatic breast cancer is the most frequent and deadliest type of cancer among young adult women [4,5], we focused on the 4T1 murine breast carcinoma model to study the efficacy of anti-cancer drug candidates. Among chemotherapeutic compounds, cisplatin, a well-known DNA crosslinker, a first line option in human carcinomas was investigated (as a reference drug) in the 4T1 murine metastatic breast carcinoma model. This type of breast carcinoma can be a relevant animal model for the human disease because (i) it is orthotopically transplanted into the mammary fat pad, (ii) it grows in immunocompetent mice (BALB/c) and (iii) gives metastasis spontaneously. Here, we studied the immunophenotype of the spleen and blood of mice with advanced cancer and with cisplatin treated cancer compared to naive mice.

We monitored the dose-response curve of cisplatin to 4T1 cells in real-time because previous studies described results with endpoint assays [41,42,43]. The half maximal inhibitory concentration (IC_50_) values showed time dependence: 36.74 µM at 24 h, 7.608 µM at 48 h, 6.962 µM at 72 h, 4.128 µM at 96 h, and 3.995 µM at 120 h (Figure 1 and Figure S1). In order to investigate the alteration of the tumor stroma or the immunophenotype changes during cancer development the 4T1 cells were orthotopically injected into BALB/c mice. Cisplatin reduced the tumor growth, the number of lung metastatic nodules, and splenomegaly (Figure 2). In addition to malignant cells, the alteration of the stroma of solid tumors, namely the activation of cancer-associated fibroblasts has been described as critical determinant of cancer development and survival, and was also confirmed as therapeutic target [2]. It was shown that the expression of FAP prolyl endopeptidase, even on the cell surface or solubilized in the plasma, correlates with the malignancy and cisplatin resistance of carcinomas [29,44,45,46]. Therefore, we have developed an assay to measure the activity of FAP cleaving the Fmoc-Gly-Pro-Cysteic acid-Ile-Gly-NH_2_ peptide substrate (Figure S2). We could show that cisplatin treatment significantly reduced the FAP activity of the plasma of 4T1 tumor bearing mice (Figure 3).

It has been widely known that the alternative polarization of the immune system, the accumulation of immature myeloid cells (MDSCs) with potent immunosuppression contribute to the cancer development [12,47]. The splenic immunophenotye of mice with advanced cancer showed the emergence of the hyaluronic acid receptor CD44+ and in a smaller extent IL-17A+ MDSCs, the loss of B220+ and CD62L+ B-cells, the loss of CD62L+ CD4+ and CD8+T-cells (Figure 5, Figure 6 and Figure 7). Myeloid expression of IL-17A in the spleen has been published as a sign of advanced cancer [26]. Cisplatin treatment could restore the splenic immunophenotype downregulating CD44+, IL-17A+ MDSCs presumably because of the smaller tumor burden, on the other hand MDSCs are also sensitive to low dose chemotherapy due to their high proliferative potential [30]. The blood showed the expression of Gr-1+^dim^, more immature MDSCs in advanced cancer (Figure 8), which has been reported to suppress T-cell proliferation and IFN-γ production [37]. The expression intensity of CD44 on circulating MDSCs was also dim suggesting more immature phenotype (Figure 8), however, the percentage of CD44+MDSCs (irrespective of the CD44 marker intensity) was higher in the blood of mice with advanced cancer (Figure 9). Cisplatin partially reduced the accumulation of CD44+/CD11+/Gr1+ myeloid cells in the circulation, but changed their phenotype via induction of INF-γ production (Figure 9). It has been reported that IFN-γ producing immature myeloid cells could exert a potent immune response against sever invasive bacterial infections [48]. Computational tools, unsupervised algorithms were used to summarize our results, such as sunburst population analysis in Figure S4 and the FlowSOM analysis in Figure S5.

The immunomodulatory effect of cisplatin by increasing tumor immunogenicity has been recently published in ovarian cancer [22]. The general overview of chemotherapeutics, especially cisplatin mediated immunomodulation has been reviewed elsewhere [49,50].

To the best of our knowledge our study is the first using single cell mass cytometry to show the immunomodulatory effect of cisplatin in the 4T1 murine model of metastatic triple negative breast cancer via downregulation of immature myeloid cells and upregulation of CD62L+ B- and T-cells and IFN-γ+ peripheral myeloid cells.

## 4. Materials and Methods

### 4.1. Real-Time Cell Electronic Sensing (RT-CES) Cytotoxicity Assay

The 4T1 cells were purchased from the ATCC (American Type Culture Collection, Manassas, VA, USA) and maintained as described previously [10]. Briefly, 4T1 were maintained in Roswell Park Memorial Institute 1640 medium (RPMI-1640) with 10% FCS. The pH of the cell culture media was controlled to be between 7.2–7.4 prior use. The medium was supplemented with 2 mM GlutaMAX, and 100 U/mL penicillin, 100 µg/mL streptomycin (Life Technologies, Carlsbad, CA, USA) before use. Cells were passed every three days and placed in a humidified incubator at 37 °C 5% CO_2_ (Sanyo, Osaka, Japan). The xCelligence real-time cell electronic sensing (RT-CES) cytotoxicity assay (Acea Biosciences, San Diego, CA, USA) was performed as previously described with some modifications [25,51]. Briefly, 96-well E-plate (Acea Biosciences) was coated with gelatin solution (0.2% in phosphate buffered saline, PBS) for 20 min at 37 °C, then gelatin was washed twice with PBS solution. Growth media (50 μL) was then gently dispensed into each well of the 96-well E-plate for background readings by the RT-CES system prior to addition of 50 μL of the cell suspension containing 2 × 10^4^ 4T1 cells. Plates were kept at room temperature in a tissue culture hood for 30 min prior to insertion into the RT-CES device in the incubator to allow cells to settle. Cell growth was monitored for 48 h by measurements of electrical impedance every 15 min. Continuous recording of impedance in cells was reflected by cell index value. 48 h later cells were treated with an increasing concentration (134 nM–100 µM) of cisplatin (Selleckchem, Houston, TX, USA), treatments are demonstrated between 3.704–100 µM. Treated and control wells were dynamically monitored over 120 h by measurements of electrical impedance every 15 min. The raw plate reads for each titration point were normalized relative to the cell index status right before treatment. Each treatment was repeated in 3 wells per plate during the experiments. The half maximal inhibitory concentration (IC_50_) was calculated with relation to untreated control cells (1 corresponds to 100% viability on the y axis), and blank wells containing media without cells. IC_50_ values (50% inhibiting concentration) were calculated by GraphPad Prism^®^ (version 5.01, La Jolla, CA, USA).

### 4.2. The 4T1 in Vivo Breast Carcinoma Model

The animal experiments were performed in accordance with animal experimentation and ethics guidelines of the EU (2010/63/EU). Experimental protocols were approved by the responsible governmental agency (National Food Chain Safety Office) in possession of an ethical clearance XXIX./128/2013.

Female Charles River-derivative BALB/c mice (8–10 week old) were purchased from Kobay Ltd., (Ankara, Turkey) and were injected orthotopically with 4T1 breast carcinoma cells (1.2 × 10^5^ cells) as described previously [9]. The animals had free access to food and water. Six mice were included into each experimental group. Treatment by cisplatin (Ebewe Pharma, Unterach am Attersee, Austria) was started after 10 days of inoculation and followed in every 5th day in 5 mg/kg dose administered intraperitoneally twice on the day of the treatment. The experiments were repeated independently two times under the same conditions, the pooled results have been presented in the paper (*n* = 12). Tumors were evaluated macroscopically by the following parameters: 1) incidence of palpable tumors was determined by the daily monitoring of animals in each experimental group; 2) tumor size was measured with a precision caliper and calculated according to the formula: d2 × D × 0.5, where d and D are the minor and major diameters, respectively; 3) after euthanizing the animals, weights of the excised primary tumors, spleens, and lungs were measured. Spleen and blood were processed freshly in order to isolate leukocytes. Mice showing signs of suffering (lost 15% of body weight and/or loss of the righting reflex and/or enable to eat, drink) due to (ethical) legislation were sacrificed.

### 4.3. Synthesis of Fmoc-Gly-Pro-Cysteic Acid-Ile-Gly-NH_2_ Peptide

In order to obtain the target peptide, Fmoc-Gly-Pro-Cysteic acid-Ile-Gly-NH_2_, first Fmoc-Gly-Pro-Cys-Ile-Gly-NH_2_ peptide was synthesized (Fmoc, 9-fluorenylmethoxycarbonyl, Avidin Ltd., Szeged, Hungary). Reagents, otherwise not stated, were purchased from Sigma-Aldrich (St. Louis, MI, USA). Fmoc-strategy synthesis was carried out manually in a solid-phase vessel on Rink Amide ChemMatrix resin, the protected Fmoc-amino acids (3 equiv.) were coupled using DCC (dicyclohexylcarbodiimide 3 equiv.) and HOBt (1-hydroxybenzotriazole, 3 equiv.) in DMF (*N,N*-dimethylformamide) for 2 h at room temperature. The deprotection of the Fmoc-group was achieved with the treatment of the resin with 5% piperazine/DMF (1 × 5 min, 1 × 20 min). Washings between coupling and deprotection were performed with DMF (3 × 1 min), MeOH (methanol) (1 × 1 min) and DMF (3 × 1 min). The completion of the coupling was monitored using the Kaiser test. Following the final coupling, the resin was washed with DMF (3 × 1 min) and MeOH (3 × 1 min), and dried under a stream of air. The dry resin was treated with TFA/H_2_O (trifluoroacetic acid/H_2_O, 95:5) for 3 h at room temperature. The cleavage mixture was lyophilized, and the pellet was redissolved in MeOH/AcN (methanol/acetonitrile, 1:1) for Liquid chromatography-mass spectrometry (LC-MS) analysis (Agilent, Santa Clara, CA, USA). The LC-MS analysis found that Fmoc-Gly-Pro-Cys-Ile-Gly-NH_2_ was obtained in 90% purity (linear gradient from 0% to 100% AcN over 30 min, t_R_: 21.77 min). LC-MS observed [M + H]^+^ 667.2, required [M + H]^+^ 667.8. The obtained Fmoc-Gly-Pro-Cys-Ile-Gly-NH_2_ was transformed into Fmoc-Gly-Pro-Cysteic acid-Ile-Gly-NH_2_ peptide with no further purification. It was dissolved in acetone, cooled to 1 °C in a mixture of ice and water. First 30% aqueous H_2_O_2_ was added, then Na_2_WO_4_.2H_2_O in catalytic amount. After 3 h, we did not detect the starting material (Fmoc-Gly-Pro-Cys-Ile-Gly-NH_2_), it completely transformed into the desired Fmoc-Gly-Pro-Cysteic acid-Ile-Gly-NH_2_ peptide. The LC-MS analysis found that Fmoc-Gly-Pro-Cysteic acid-Ile-Gly-NH_2_ was obtained in 75% purity (linear gradient from 0% to 100% AcN over 30 min, t_R_: 16.90 min). LC-MS observed [M + H]^+^ 715.2, required [M + H]^+^ 715.8. To purify the crude peptide, it was dissolved in AcN/MeOH/H_2_O, then filtered, using a 0.45 μm nylon filter. Gradient elution was used, 70–100% AcN in 60 min at a 3 mL min-1 flow rate with detection at 220 nm. Pure fractions were collected and lyophilized to give a fluffy white material.

### 4.4. FAP Activity Assay

Blood from control and tumor bearing (untreated and cisplatin treated) BALB/c mice was drawn (200 µL) into EDTA containing tubes (separately from 3 animals from each group). Reagents, otherwise not stated, were purchased from Sigma-Aldrich. Blood samples were centrifuged at 12,000 RPM for 10 min. Supernatant (plasma) was removed and transferred to fresh tubes and were immediately used. Digestion reactions were set up by combining 36 µL plasma and 9 µL solution of cysteine acid containing peptide solution (5 mg/mL) for a final peptide concentration of 1 mg/mL. Reactions were incubated at 37 °C for 60 min. After incubation, 75 µL of acetonitrile containing 0.2% trifluoro-acetic (TFA) acid was added and samples were centrifuged at 12,000 RPM for 5 min. 80 µL of supernatant was removed and transferred to fresh tubes and were stored at −20 °C until analysis. Area under the curve (AUC) values of the peptide digestion product **2** were plotted.

### 4.5. Mass Cytometry

Single cell mass cytometry (CyTOF, Fluidigm, San Francisco, CA, USA)) was performed as described previously with some modifications [52]. Briefly, naive, 4T1 breast tumor bearing and cisplatin treated 4T1 tumor bearing mice were euthanized on the 23rd day after 4T1 injection. Spleen and blood were processed freshly. Withdrawal of the blood was carried by cardiac puncture using 50 μL EDTA (30 mg/mL) per syringe (Beckton Dickinson, Franklin Lakes, NY, USA). Spleen was smashed on 100 µm cell strainer (VWR, Radnoe, PA, USA), washed with PBS and centrifuged at 1400 rpm 5 min. Blood was centrifuged at 2000 rpm for 10 min, plasma was harvested and stored at −80 °C. Both the pellet of spleen and blood were resuspended. Red blood cell lysis was carried out by the incubation of cells with 5 mL ACK (0.155 M NH_4_Cl, 10 mM KHCO_3_, 0.1 mM Na_2_EDTA, pH 7.3, Sigma-Aldrich) solution for 5 min. Samples were loaded on cell strainer (70 μm in pore size,) and washed by 20 mL PBS. Cells were counted using Bürker chamber and trypan blue viability dye. Three million cells were pooled from six mice per group for mass cytometry (Helios, Fluidigm, San Francisco, CA, USA). The in vivo experiment and CyTOF were repeated twice. Cells viability was determined by cisplatin (5 μM 195Pt, Fluidigm) staining for 3 min on ice in 300 μL PBS. Sample was diluted by 1500 μL Maxpar Cell Staining Buffer (MCSB, Fluidigm) and centrifuged at 350 g for 5 min. Cells were suspended in 50 μL MCSB and the antibody mix (Table 1) was added in 50 μL (each antibody diluted finally 1:100). Two commercially available antibody panel was combined, the Maxpar^®^ Mouse Sp/LN Phenotyping Panel kit (Fluidigm, cat. numbers 201306) and Maxpar^®^ Mouse Intracellular I Cytokine Panel kit (Fluidigm, cat. number 201310).

Samples after 60 min incubation at 4 °C, antibodies were washed by 2 mL MCSB and centrifuged at 300 g 5 min, two times. The pellet was suspended in the residual volume. Cells were fixed in 1.6% formaldehyde (freshly diluted from 16% Pierce formaldehyde with PBS, Thermo Fisher Scientific, Waltham, MA, USA) and incubated for 10 min at room temperature. Cells were centrifuged at 800 g for 5 min. Cell ID DNA intercalator (125 µM, 191/193 Iridium, Fluidigm) was added in 1000× dilution in Maxpar Fix and Perm for overnight at 4 °C. Cells for the acquisition were centrifuged at 800g for 5 min then were washed by 2 mL MCSB and centrifuged at 800 g for 5 min. Cells were suspended in 1 mL PBS (for WB injector) and counted in Bürker-chamber during centrifugation. For the acquisition the concentration of cells was set to 0.5 × 10^6^/mL in cell acquisition solution (CAS, Fluidigm) containing 10% EQ Calibration Beads. Cells were filtered through 30 μm Celltrics gravity filter (Sysmex, Görlitz, Germany) and acquired freshly. Mass cytometry data were analyzed in Cytobank (Beckman Coulter, Brea, CA, USA). Single living cells were determined. The viSNE analysis was carried-out (iterations = 1000, perplexity = 30, theta = 0.5) on 5 × 10^4^ for the spleen and 2 × 10^4^ for blood of CD45+ living singlets. Reduction of dimensionality was performed by FlowSOM also, by an algorithm creating self-organizing maps during automated clustering in Cytobank [35].

### 4.6. Statistical Analysis

Results are shown as arithmetic mean ± standard error of the mean (SEM); statistical comparisons were performed by two-tailed Student’s t-test as pairwise comparisons as described in the figure legends. In all statistical comparisons, probability “*p*” was set as the level of significance (set at * *p* < 0.05, ** *p* < 0.01, ***, *p* < 0.001). Data were processed and analyzed using Microsoft Excel (Microsoft Office 2016, Redmond, WA, USA), and visualized using GraphPad Prism or Cytobank.

## 5. Conclusions

Our findings showed that cisplatin treatment reduced tumor growth, number of lung metastasis and the splenomegaly of 4T1 tumor bearing mice. Cisplatin inhibited the tumor stroma formation, the activation of carcinoma-associated fibroblasts by the diminished proteolytic activity of fibroblast activating protein. Single cell mass cytometry revealed that cisplatin could exert a potent immunomodulatory effect via inhibiting the accumulation of splenic MDSCs in a murine model of metastatic triple negative breast cancer. Emergence of certain myeloid subsets in the spleen, such as CD44+MDSCs and IL-17A+MDSC were associated with advanced cancer, while within the lymphoid subsets, the absence the B220+ B-cells, CD62L+ B-cells, CD62L+CD4+ T-cells, and CD62L+ CD8+ T-cells was shown in the untreated tumor bearing mice. However, cisplatin treatment could restore the splenic immunophenotype similar to naive mice. Peripheral MDSCs in the circulation were not completely eliminated by cisplatin but myeloid-derived IFN-γ production was increased. Thus, our study highlights the use of low-dose chemotherapy, such as cisplatin in combination with immunotherapies to treat triple negative breast cancer.

## Figures and Tables

**Table 1:** The list of the antibodies used for mass cytometry.

Target	Clone	Metal Tag
Gr-1 (Ly6C/Ly6G)	RB6-8C5	141_Pr
CD11c	N418	142_Nd
CD69	H1.2F3	145_Nd
CD45	30-F11	147_Sm
CD11b	M1/70	148_Nd
CD19	6D5	149_Sm
CD25	3C7	151_Eu
CD3e	145-2C11	152_Sm
TER-119	TER119	154_Sm
CD62L	MEL-14	160_Gd
CD8a	53-6.7	168_Er
TCRβ	H57-597	169_Tm
NK1.1	PK136	170_Er
CD44	IM7	171_Yb
CD4	RM4-5	172_Yb
B220	Ra3-6B2	176_Yb
IFN-γ	XMG1.2	165_Ho
IL-2	JES6-5H4	144_Nd
IL-4	11B11	166_Er
IL-5	TRFK5	143_Nd
IL-6	MP5-20F3	167_Er
IL-10	JES5-16E3	158_Gd
IL-17A	TC11-18H10.1	174_Yb
TNFα	MP6-XT22	162_Dy

## Supplementary Materials

Supplementary Materials can be found at https://www.mdpi.com/1422-0067/21/1/170/s1.
