# Supplementary material for: Single Cell Mass Cytometry Revealed the Immunomodulatory Effect of Cisplatin Via Downregulation of Splenic CD44+, IL-17A+ MDSCs and Promotion of Circulating IFN-γ+ Myeloid Cells in the 4T1 Metastatic Breast Cancer Model
Source: Int J Mol Sci. 2019 Dec 25;21(1):170. doi: 10.3390/ijms21010170 (PMC6982301; doi:10.3390/ijms21010170)

Figure S1. Dose-response curves of cisplatin treated 4T1 cells using xCelligence real-time electronic sensing assay

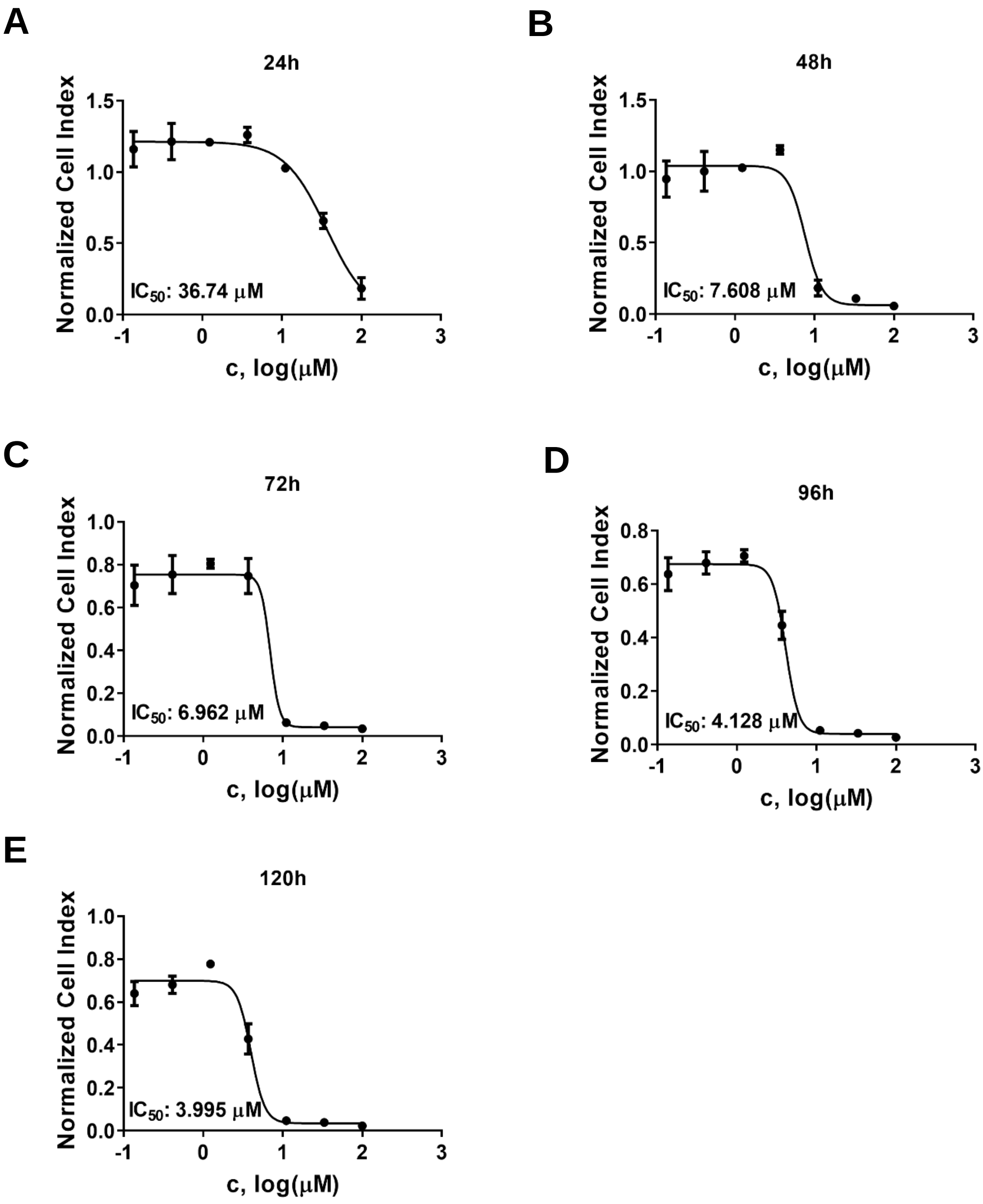

Supplement: Supplementary file 1 [file ijms-21-00170-s001.zip › Rev_supplement/Figure_S1.pdf]
