# Supplementary material for: Single Cell Mass Cytometry Revealed the Immunomodulatory Effect of Cisplatin Via Downregulation of Splenic CD44+, IL-17A+ MDSCs and Promotion of Circulating IFN-γ+ Myeloid Cells in the 4T1 Metastatic Breast Cancer Model
Source: Int J Mol Sci. 2019 Dec 25;21(1):170. doi: 10.3390/ijms21010170 (PMC6982301; doi:10.3390/ijms21010170)

CD4+ T-cells

Figure S3. Manual gating strategy of spleen -derived leukocytes of naive, 4T1 tumor bearing and cisplatin treated 4T1 tumorous mice

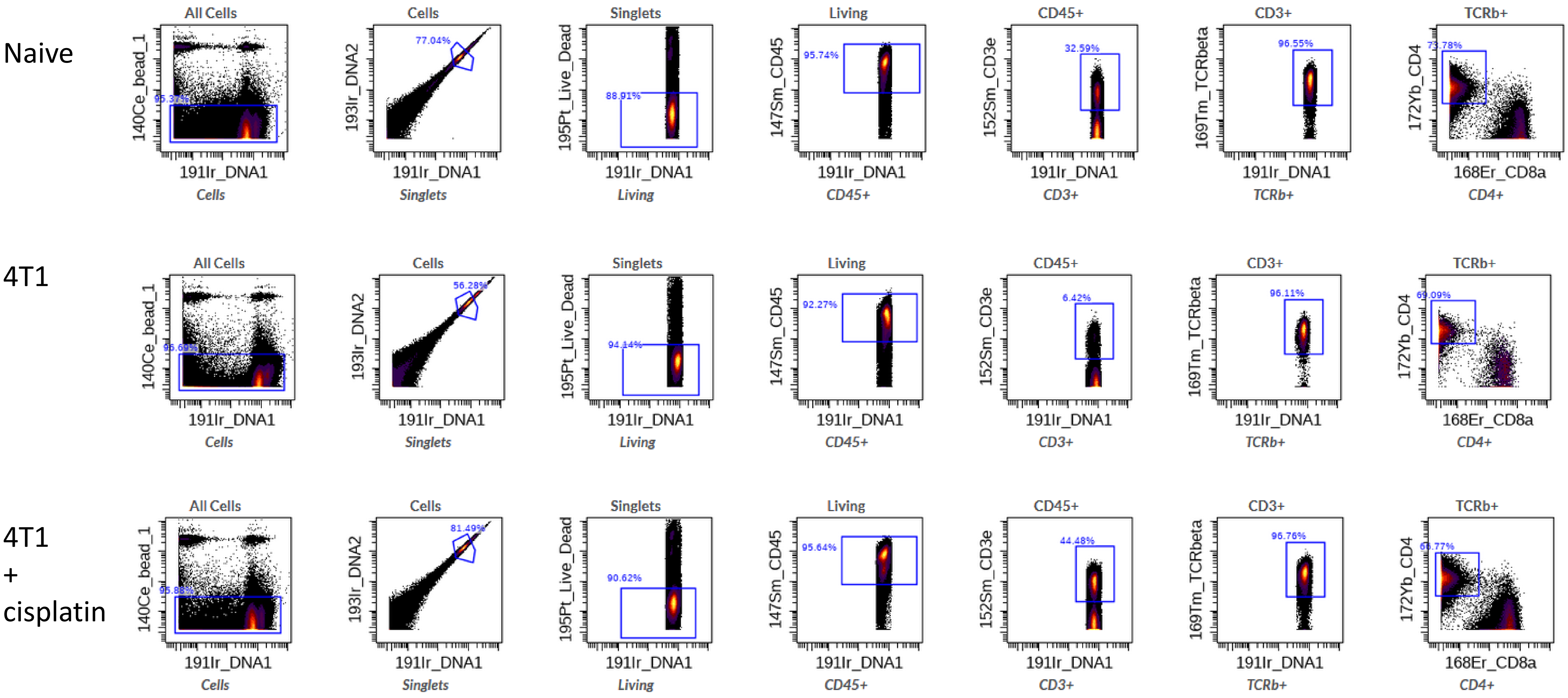

CD8+ T-cells

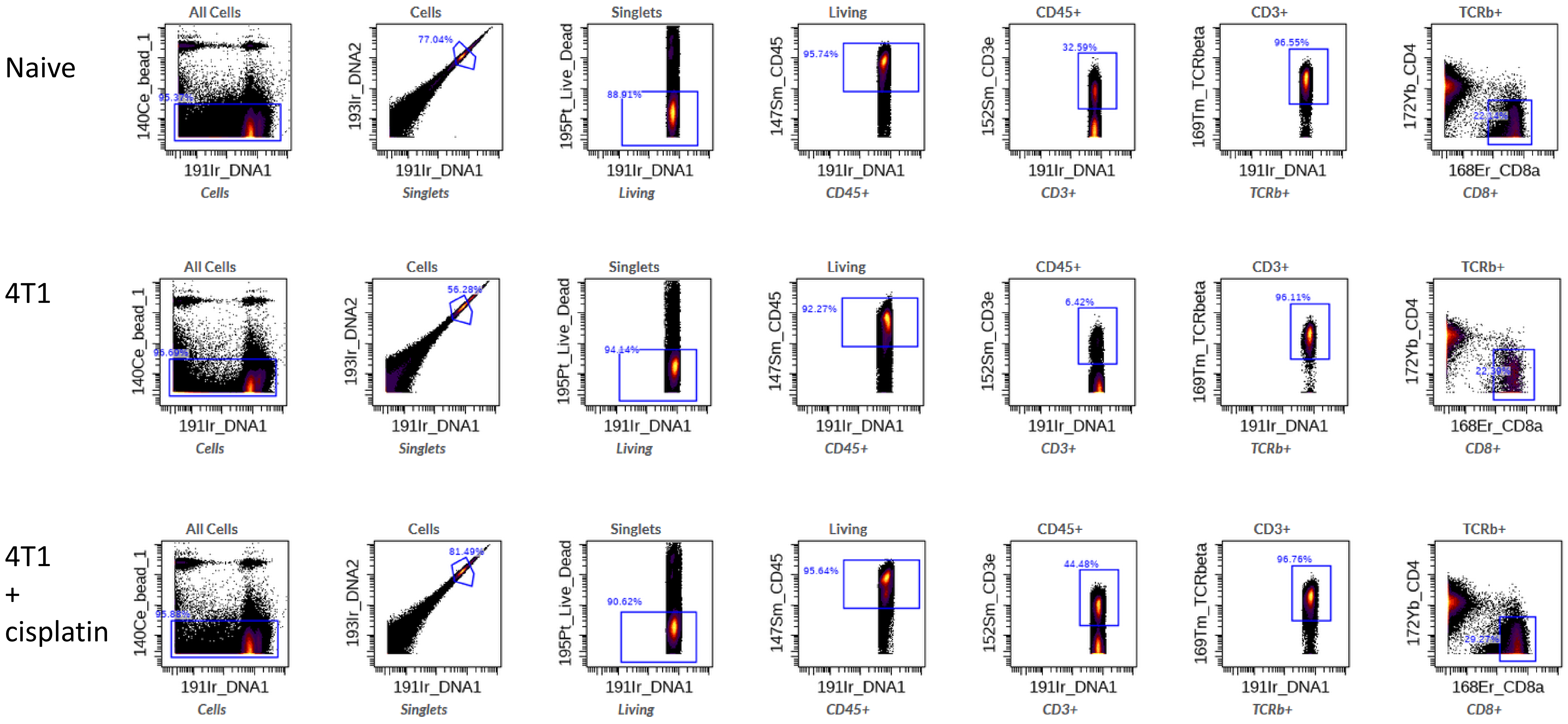
Naive
4T1
4T1
+
cisplatin
All Cells
Cells
Singlets
Living
CD45+
CD3+
TCRb+
140Ce_bead_1
193Ir_DNA2
195Pt_Live_Dead
147Sm_CD45
152Sm_CD3e
169Tm_TCRbeta
172Yb_CD4
191Ir_DNA1
168Er_CD8a
CD8+
77.04%
88.91%
95.74%
32.59%
96.55%
56.28%
94.14%
92.27%
6.42%
96.11%
81.49%
90.62%
95.64%
44.48%
96.76%

CD19+ B-cells

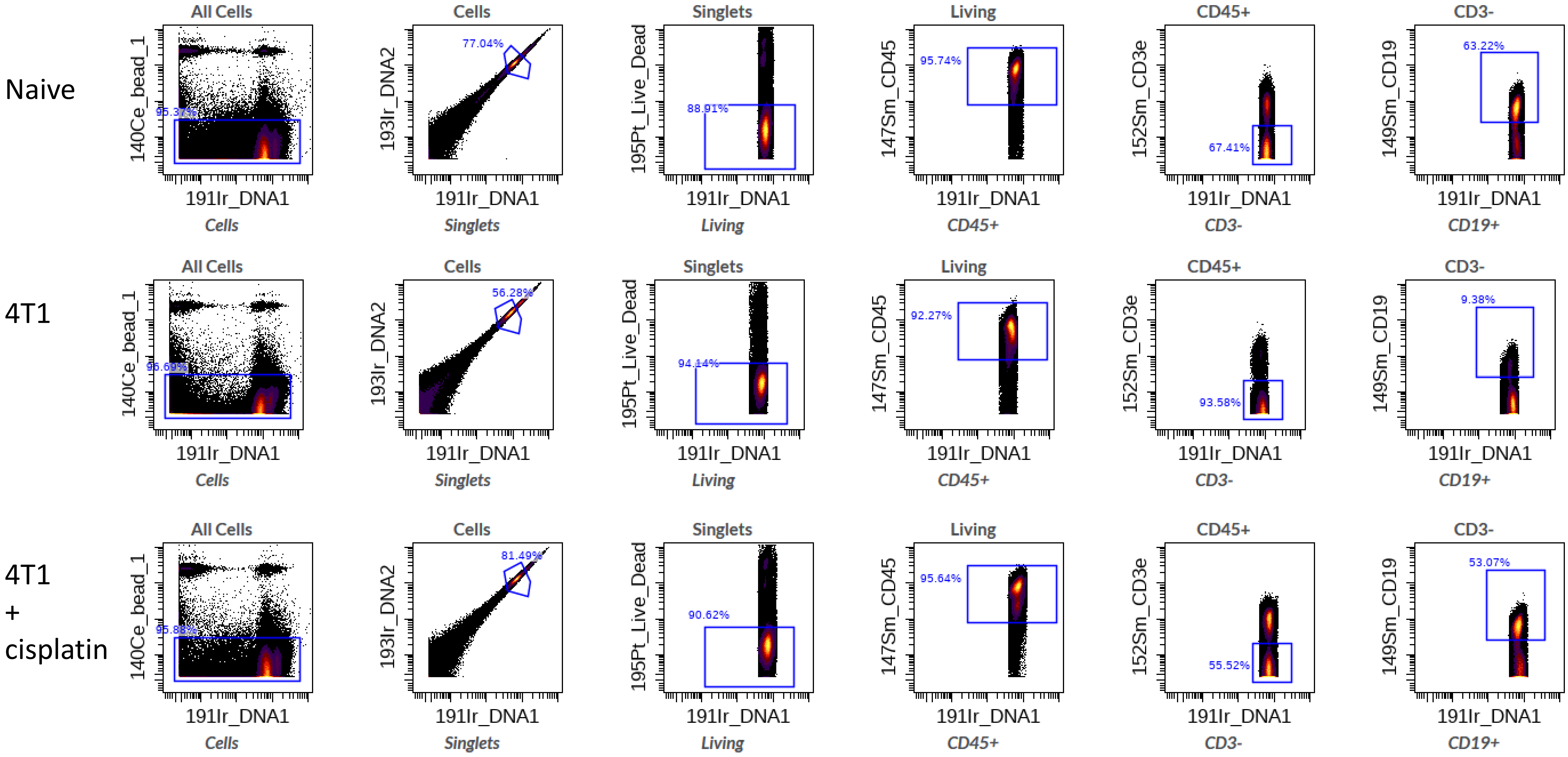
Naive
4T1
4T1
+
cisplatin
All Cells
Cells
Singlets
Living
CD45+
CD3-
140Ce_bead_1
193Ir_DNA2
195Pt_Live_Dead
147Sm_CD45
152Sm_CD3e
149Sm_CD19
191Ir_DNA1
CD19+
95.37%
77.04%
88.91%
95.74%
67.41%
63.22%
96.69%
56.28%
94.14%
92.27%
93.58%
9.38%
95.88%
81.49%
90.62%
95.64%
55.52%
53.07%

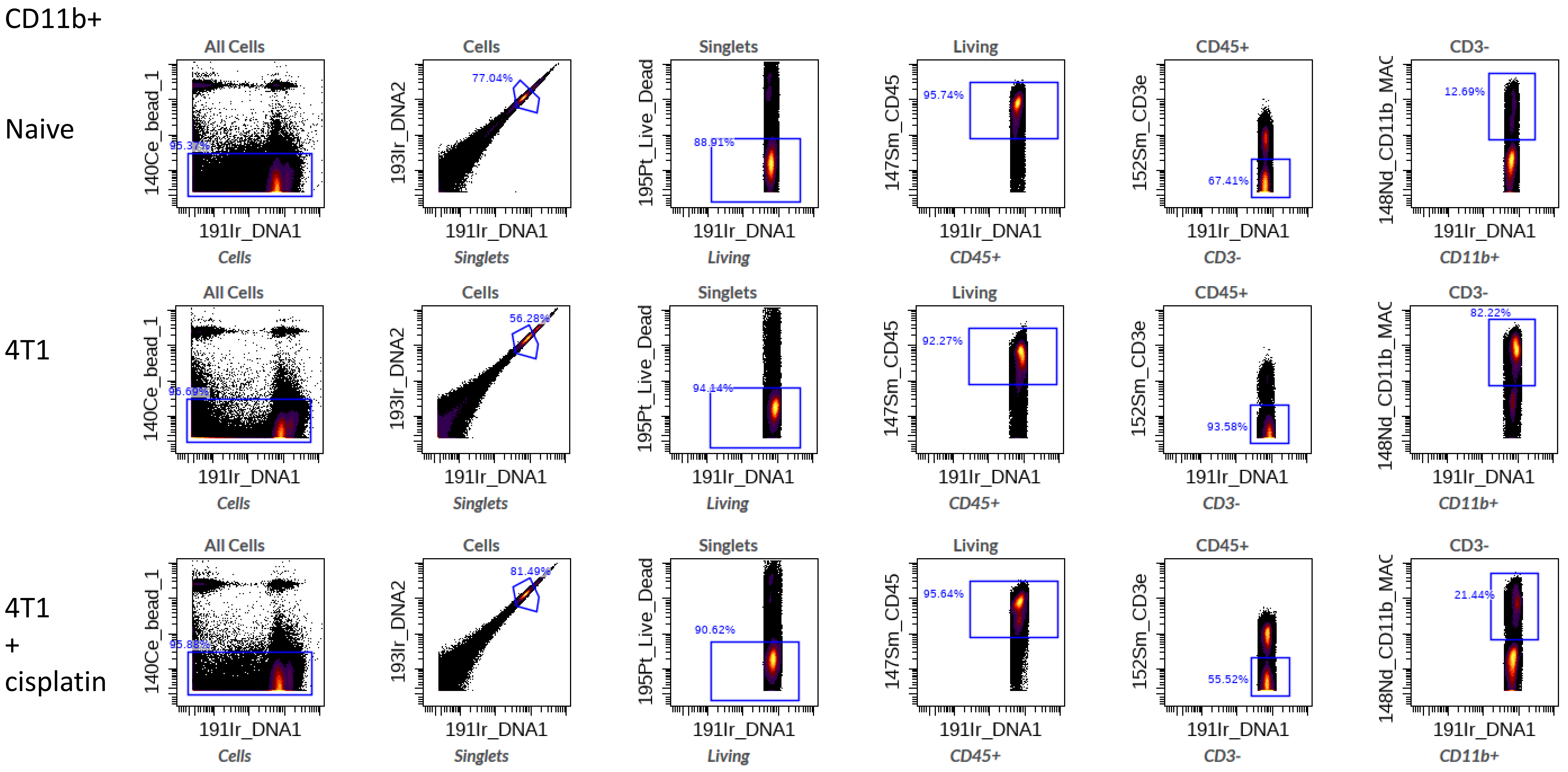
CD11b+
Naive
4T1
4T1
+
cisplatin
All Cells
Cells
Singlets
Living
CD45+
CD3-
140Ce_bead_1
193Ir_DNA2
195Pt_Live_Dead
147Sm_CD45
152Sm_CD3e
148Nd_CD11b_MAC
191Ir_DNA1
Cells
Singlets
Living
CD45+
CD3-
CD11b+
95.37%
77.04%
88.91%
95.74%
67.41%
12.69%
96.69%
56.28%
94.14%
92.27%
93.58%
82.22%
95.88%
81.49%
90.62%
95.64%
55.52%
21.44%

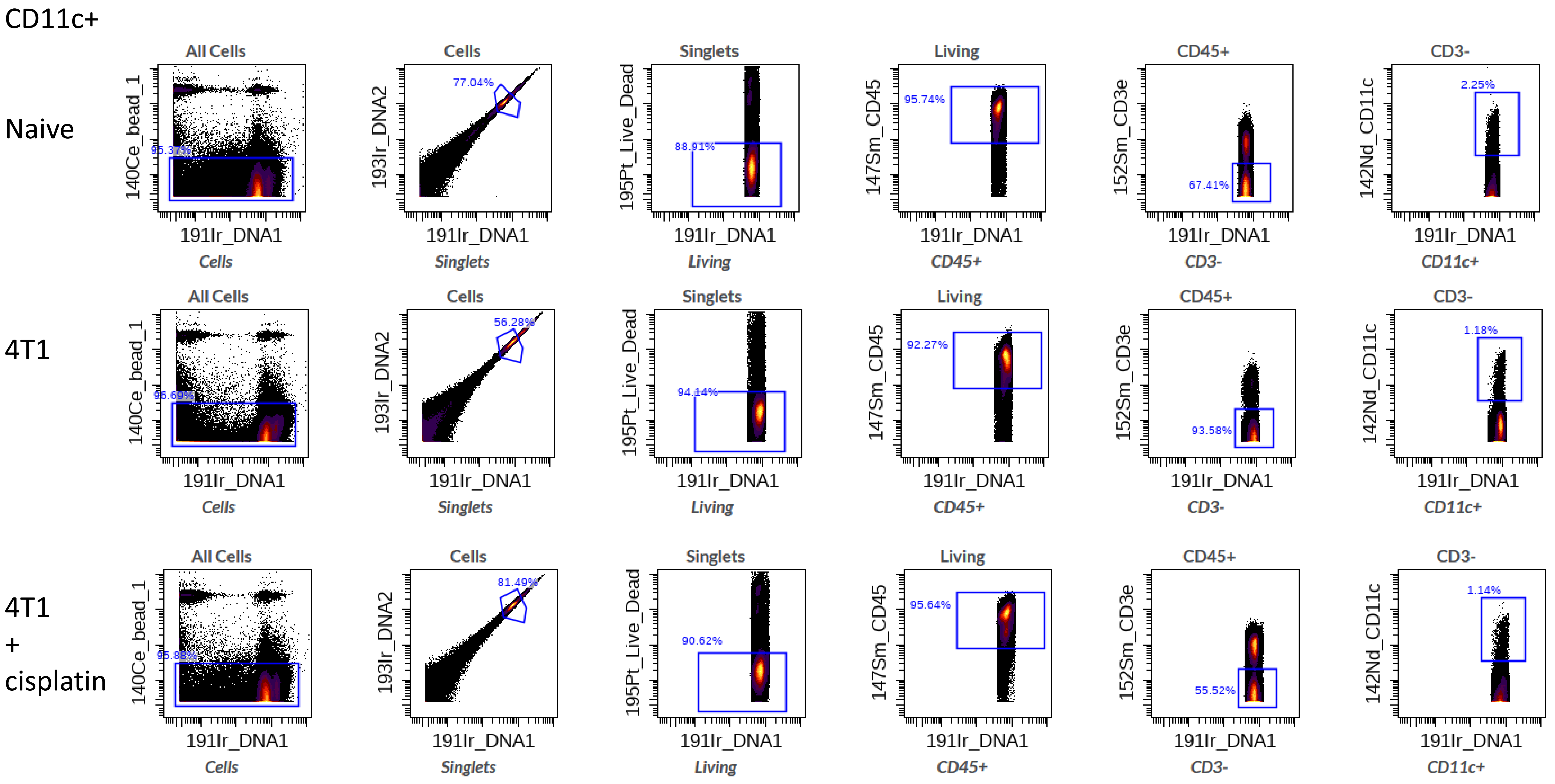

CD11c+
Naive
4T1
4T1
+
cisplatin
All Cells
Cells
Singlets
Living
CD45+
CD3-
140Ce_bead_1
193Ir_DNA2
195Pt_Live_Dead
147Sm_CD45
152Sm_CD3e
142Nd_CD11c
191Ir_DNA1
CD11c+
95.37%
77.04%
88.91%
95.74%
67.41%
2.25%
96.69%
56.28%
94.14%
92.27%
93.58%
1.18%
95.88%
81.49%
90.62%
95.64%
55.52%
1.14%

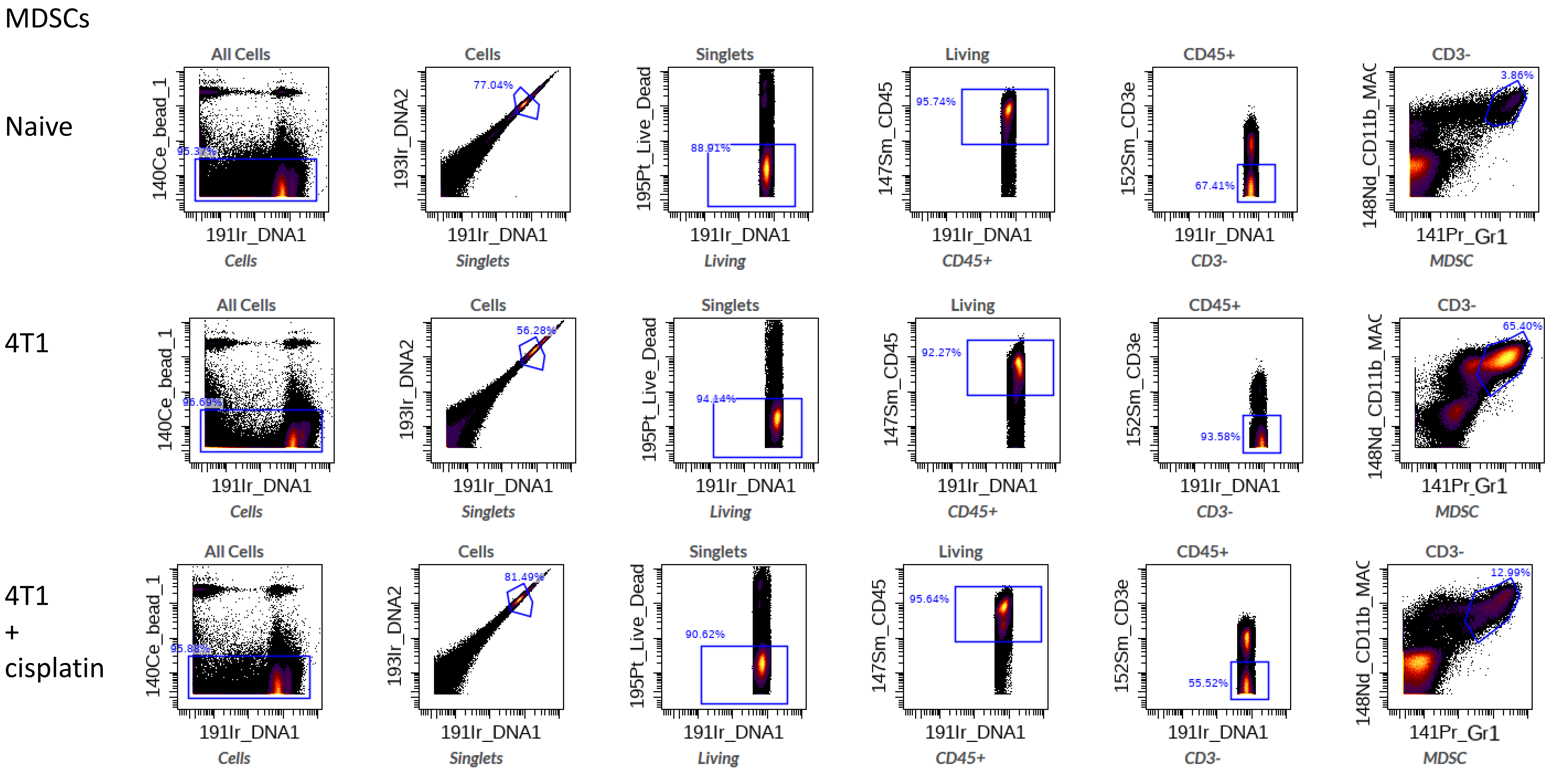
MDSCs
Naive
All Cells
140Ce_bead_1
95.37%
191Ir_DNA1
Cells
193Ir_DNA2
77.04%
Singlets
195Pt_Live_Dead
88.91%
Living
147Sm_CD45
95.74%
CD45+
152Sm_CD3e
67.41%
CD3-
148Nd_CD11b_MAC
3.86%
141Pr_Gr1
MDSC
4T1
All Cells
140Ce_bead_1
96.69%
191Ir_DNA1
Cells
193Ir_DNA2
56.28%
Singlets
195Pt_Live_Dead
94.14%
Living
147Sm_CD45
92.27%
CD45+
152Sm_CD3e
93.58%
CD3-
148Nd_CD11b_MAC
65.40%
141Pr_Gr1
MDSC
4T1
+
cisplatin
All Cells
140Ce_bead_1
95.88%
191Ir_DNA1
Cells
193Ir_DNA2
81.49%
Singlets
195Pt_Live_Dead
90.62%
Living
147Sm_CD45
95.64%
CD45+
152Sm_CD3e
55.52%
CD3-
148Nd_CD11b_MAC
12.99%
141Pr_Gr1
MDSC

CD44+ MDSCs

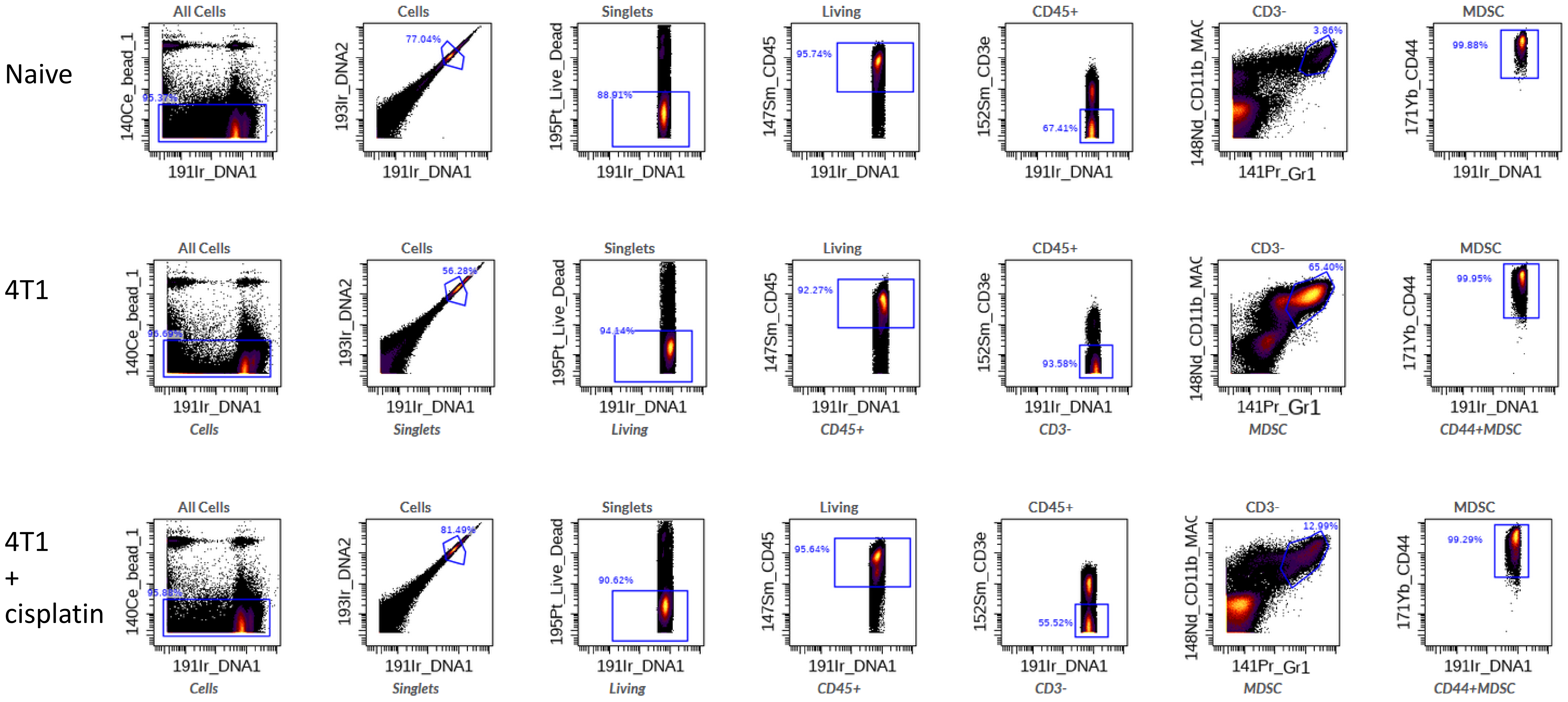

Naive
4T1
4T1 + cisplatin
All Cells
Cells
Singlets
Living
CD45+
CD3-
MDSC
140Ce_bead_1
193Ir_DNA2
195Pt_Live_Dead
147Sm_CD45
152Sm_CD3e
148Nd_CD11b_MAC
171Yb_CD44
191Ir_DNA1
141Pr_Gr1
77.04%
88.91%
95.74%
67.41%
3.86%
99.88%
56.28%
94.14%
92.27%
93.58%
65.40%
99.95%
81.49%
90.62%
95.64%
55.52%
12.99%
99.29%
CD44+MDSC

IL-17A+ MDSCs

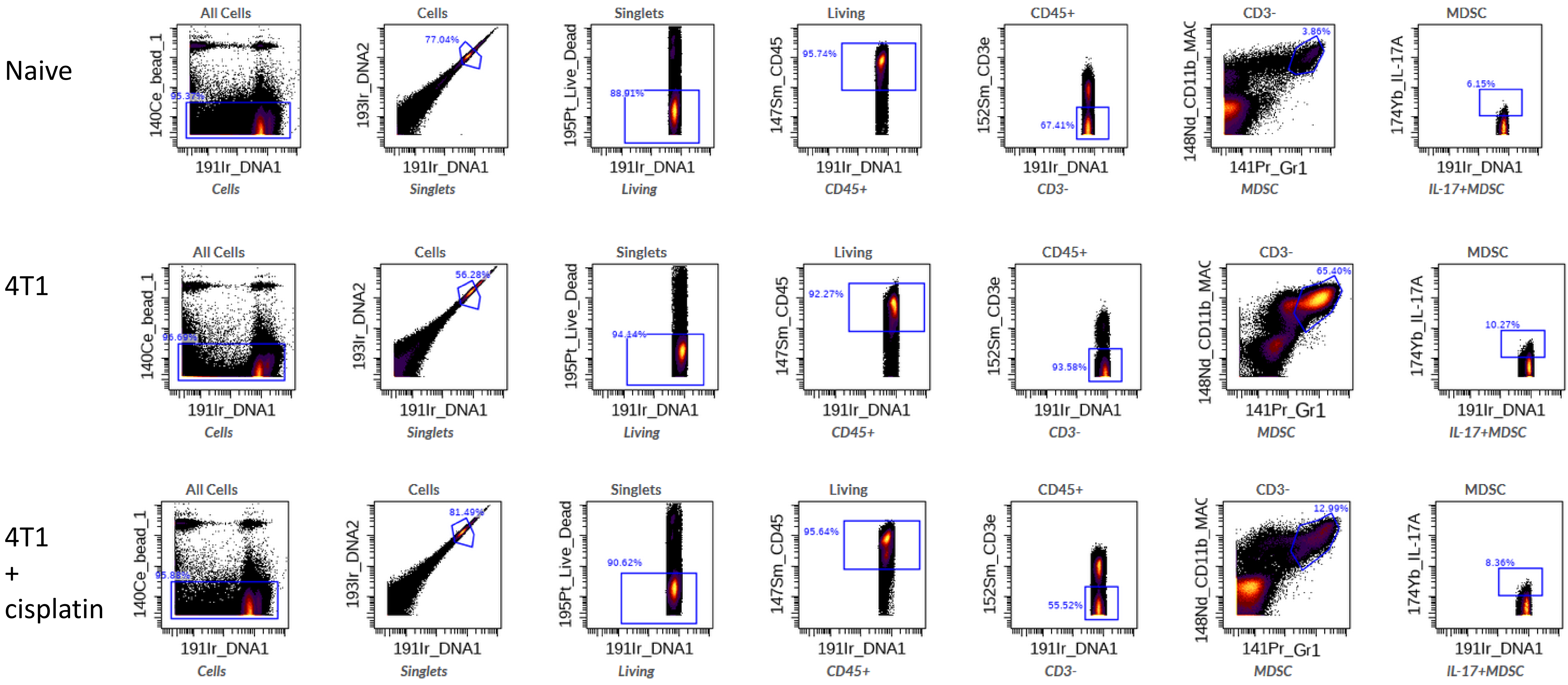

Naive
4T1
4T1
+
cisplatin
All Cells
Cells
Singlets
Living
CD45+
CD3-
MDSC
140Ce_bead_1
193Ir_DNA2
195Pt_Live_Dead
147Sm_CD45
152Sm_CD3e
148Nd_CD11b_MAC
174Yb_IL-17A
191Ir_DNA1
141Pr_Gr1
Cells
Singlets
Living
CD45+
CD3-
MDSC
IL-17+MDSC
95.37%
77.04%
88.91%
95.74%
67.41%
3.86%
6.15%
96.69%
56.28%
94.14%
92.27%
93.58%
65.40%
10.27%
95.88%
81.49%
90.62%
95.64%
55.52%
12.99%
8.36%

B220+ B-cells

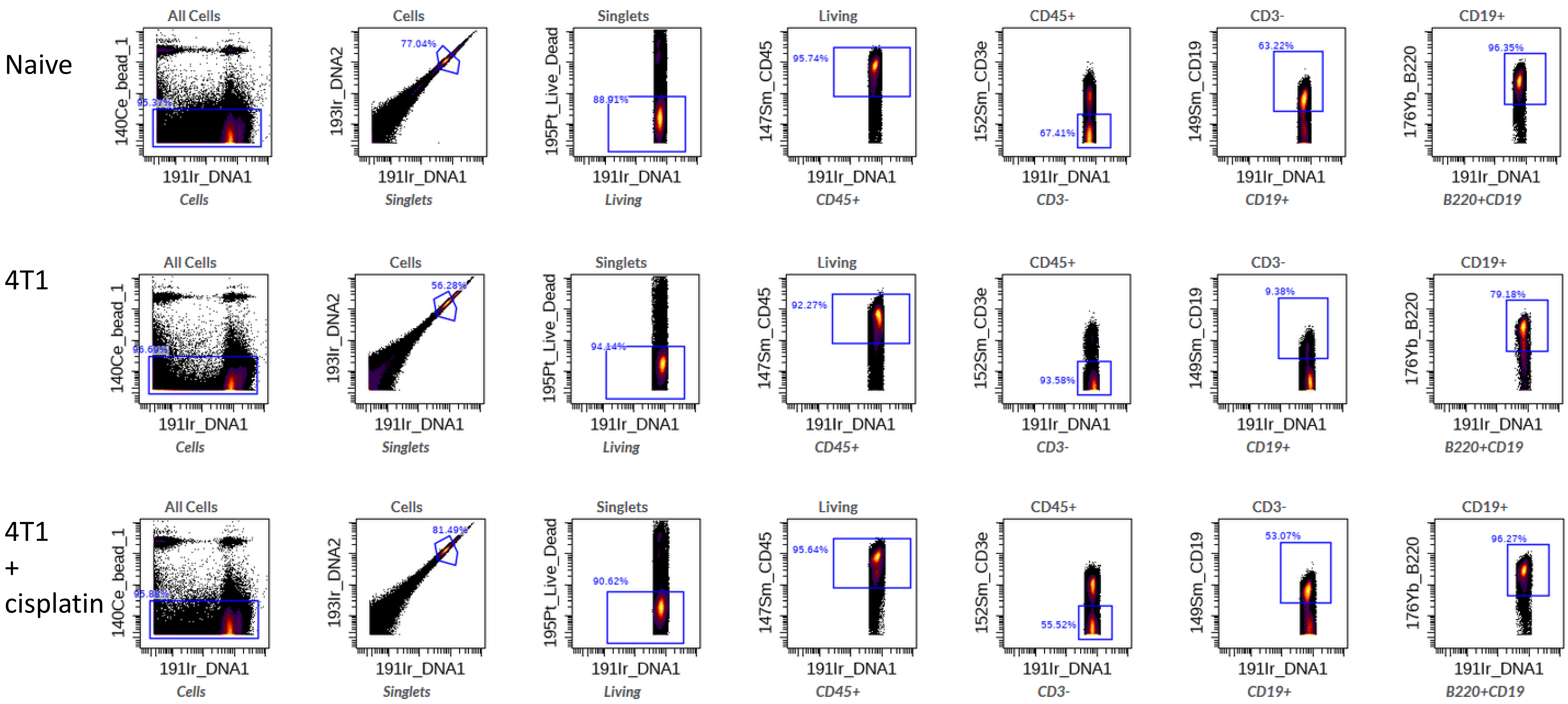

Naive
All Cells
Cells
Singlets
Living
CD45+
CD3-
CD19+
140Ce_bead_1
193Ir_DNA2
195Pt_Live_Dead
147Sm_CD45
152Sm_CD3e
149Sm_CD19
176Yb_B220
191Ir_DNA1
95.37%
77.04%
88.91%
95.74%
67.41%
63.22%
96.35%
Cells
Singlets
Living
CD45+
CD3-
CD19+
B220+CD19
4T1
96.69%
56.28%
94.14%
92.27%
93.58%
9.38%
79.18%
4T1
+
cisplatin
95.88%
81.49%
90.62%
95.64%
55.52%
53.07%
96.27%

CD62L+ B-cells

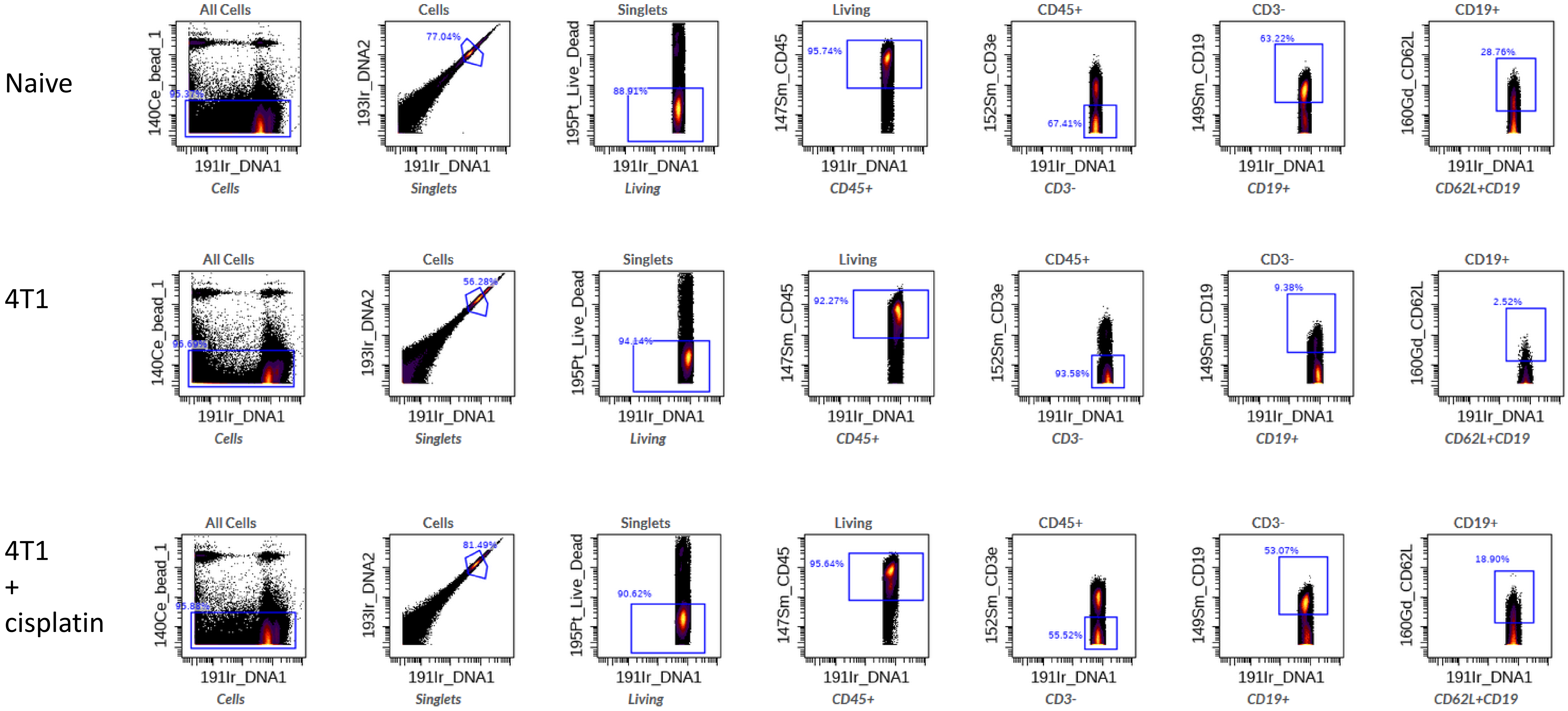

CD62L+ CD4+ T-cells

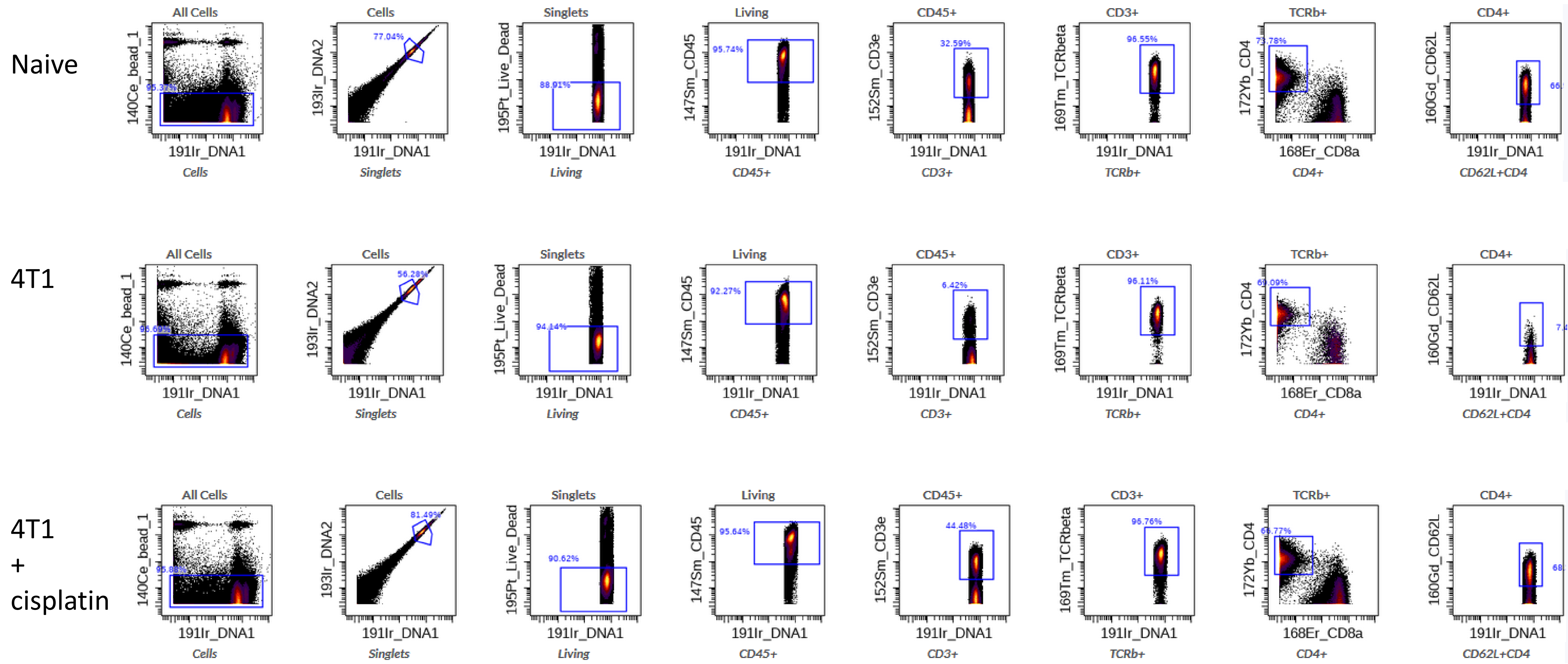

CD62L+ CD8+ T-cells

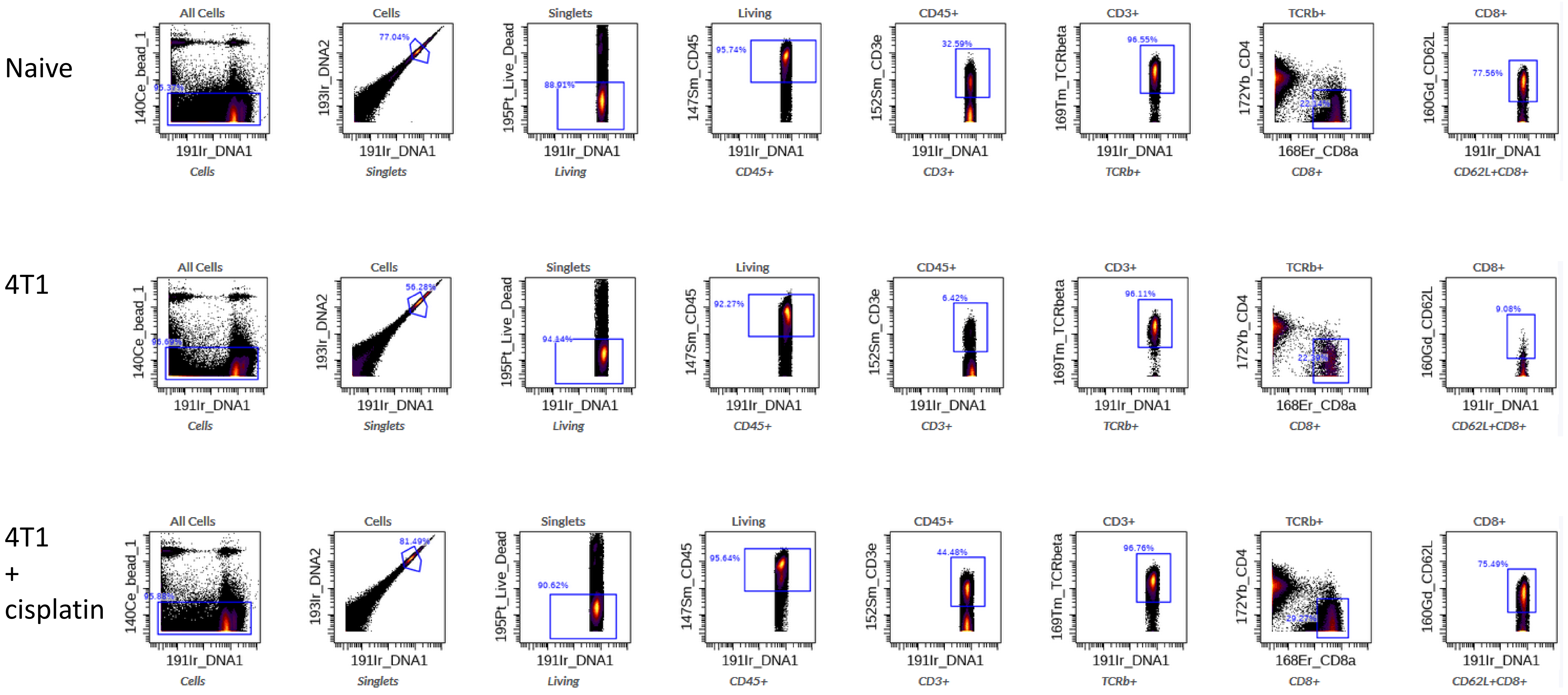

Supplement: Supplementary file 1 [file ijms-21-00170-s001.zip › Rev_supplement/Figure_S3.pdf]
