# Supplementary material for: Single Cell Mass Cytometry Revealed the Immunomodulatory Effect of Cisplatin Via Downregulation of Splenic CD44+, IL-17A+ MDSCs and Promotion of Circulating IFN-γ+ Myeloid Cells in the 4T1 Metastatic Breast Cancer Model
Source: Int J Mol Sci. 2019 Dec 25;21(1):170. doi: 10.3390/ijms21010170 (PMC6982301; doi:10.3390/ijms21010170)
Supplement: Supplementary file 1 [file ijms-21-00170-s001.zip › Rev_supplement/Supplementary Figure Legend.docx]

Supplementary Figure Legend

**Figure S1.** Dose-response curves of cisplatin treated 4T1 cells using xCelligence real-time electronic sensing assay**.** Half maximal inhibitory concenrations (IC_50_) of cisplatin on the viability of 4T1 cells was determined on a time dependent manner. After 48 h of cell seeding cisplatin was added (137 nM-100 µM) and viability was monitored for 120 h in every 15 minutes as described in the Materials and Methods 4.1.

**Figure S2.** Schematic illustration of FAP digestion of Fmoc-Gly-Pro-Cysteic acid-Ile-Gly-NH2 peptide (**1**). The peptide substrate (**1**) was synthetized in our laboratory as described in the Materials and Methods 4.3. The activity of FAP was measured from the plasma samples of naive, 4T1 tumor bearing and cisplatin treated 4T1 tumorous mice as described in the Materials and Methods 4.4. Area under the curve (AUC) values from HPLC analysis of the peptide digestion product (**2**) was measured.

**Figure S3.** Manual gating of spleen-derived leukocytes of naive, 4T1 tumor bearing and cisplatin treated 4T1 tumorous mice. Manual gating was performed in Cytobank. This Cytobank report shows the percentage of cells in one given population in relation to the parental population. However, populations were quantified in the main text in relation to the CD45+ living singlets from the data of numerical report of Cytobank.

**Figure S4.** Sunburst charts represent the immunocomposition of main subsets detected by single cell mass cytometry of the spleen (A-C) and blood-derived leukocytes (D-E) in naive, 4T1 tumor bearing and cisplatin treated 4T1 tumorous mice. Single cell mass cytometry was carried out with 24 antibodies as described in the Materials and Methods 4.5. Manual gating and reporting of sunburst charts were performed within the CD45+ living singlets in Cytobank.

**Figure S5.** FlowSOM (Flow data of Self-Organizing Maps) analysis of spleen and blood-derived leukocytes of naive, 4T1 tumor bearing and cisplatin treated 4T1 tumor bearing samples. Reduction of dimensionality was performed by FlowSOM, an algorithm creating Minimum Spanning Trees (MSTs) during automated, unsupervised clustering in Cytobank. Similar cells are assigned to the same node, the size of pie charts corresponds to the number of events within that cluster, main subsets are highlighted in the graph.
